# Downregulation of Glutamine Synthetase, not glutaminolysis, is responsible for glutamine addiction in Notch1‐driven acute lymphoblastic leukemia

**DOI:** 10.1002/1878-0261.12877

**Published:** 2021-02-13

**Authors:** Tra Ly Nguyen, Marie‐Julie Nokin, Silvia Terés, Mercedes Tomé, Clément Bodineau, Oriane Galmar, Jean‐Max Pasquet, Benoit Rousseau, Sebastian van Liempd, Juan Manuel Falcon‐Perez, Elodie Richard, Elodie Muzotte, Hamid‐Reza Rezvani, Muriel Priault, Marion Bouchecareilh, Isabelle Redonnet‐Vernhet, Julien Calvo, Benjamin Uzan, Françoise Pflumio, Patricia Fuentes, Maria L. Toribio, Abdel‐Majid Khatib, Pierre Soubeyran, Piedad del Socorro Murdoch, Raúl V. Durán

**Affiliations:** ^1^ Institut Européen de Chimie et Biologie INSERM U1218 Université de Bordeaux Pessac France; ^2^ Centro Andaluz de Biología Molecular y Medicina Regenerativa ‐ CABIMER, Consejo Superior de Investigaciones Científicas Universidad de Sevilla, Universidad Pablo de Olavide Seville Spain; ^3^ Angiogenesis and Cancer Microenvironment Laboratory INSERM U1029 Université de Bordeaux Pessac France; ^4^ INSERM BMGIC, U1035 University of Bordeaux France; ^5^ Service Commun des Animaleries University of Bordeaux France; ^6^ Exosomes Laboratory and Platform of Metabolomics CIC bioGUNE CIBERehd Derio Spain; ^7^ IKERBASQUE Basque Foundation for Science Bilbao Spain; ^8^ Institut Bergonié INSERM U1218 University of Bordeaux France; ^9^ Institut de Biochimie et Génétique Cellulaires CNRS UMR 5095 Université de Bordeaux France; ^10^ Bordeaux Research in Translational Oncology INSERM U1053 Université de Bordeaux France; ^11^ Maladies Héréditaires du Métabolisme Laboratoire de Biochimie Hôpital Pellegrin CHU Bordeaux France; ^12^ UMR967 Inserm CEA Université Paris 7 Université Paris 11 Fontenay‐aux‐Roses France; ^13^ Centro de Biología Molecular “Severo Ochoa” Consejo Superior de Investigaciones Científicas Universidad Autónoma de Madrid Spain; ^14^ Departamento de Bioquímica Vegetal y Biología Molecular Universidad de Sevilla Spain

**Keywords:** glutamine, glutamine synthetase, metabolic addiction, mTORC1, Notch1, T‐cell acute lymphoblastic leukemia

## Abstract

The cellular receptor Notch1 is a central regulator of T‐cell development, and as a consequence, Notch1 pathway appears upregulated in > 65% of the cases of T‐cell acute lymphoblastic leukemia (T‐ALL). However, strategies targeting Notch1 signaling render only modest results in the clinic due to treatment resistance and severe side effects. While many investigations reported the different aspects of tumor cell growth and leukemia progression controlled by Notch1, less is known regarding the modifications of cellular metabolism induced by Notch1 upregulation in T‐ALL. Previously, glutaminolysis inhibition has been proposed to synergize with anti‐Notch therapies in T‐ALL models. In this work, we report that Notch1 upregulation in T‐ALL induced a change in the metabolism of the important amino acid glutamine, preventing glutamine synthesis through the downregulation of glutamine synthetase (GS). Downregulation of GS was responsible for glutamine addiction in Notch1‐driven T‐ALL both *in vitro* and *in vivo*. Our results also confirmed an increase in glutaminolysis mediated by Notch1. Increased glutaminolysis resulted in the activation of the mammalian target of rapamycin complex 1 (mTORC1) pathway, a central controller of cell growth. However, glutaminolysis did not play any role in Notch1‐induced glutamine addiction. Finally, the combined treatment targeting mTORC1 and limiting glutamine availability had a synergistic effect to induce apoptosis and to prevent Notch1‐driven leukemia progression. Our results placed glutamine limitation and mTORC1 inhibition as a potential therapy against Notch1‐driven leukemia.

## Graphical abstract

During glutamine sufficiency, both Notch‐positive and Notch‐negative T‐ALL cells promote glutamine catabolism, leading to mammalian target of rapamycin complex 1 (mTORC1) activation and cell growth and proliferation. However, during glutamine scarcity, only Notch‐negative T‐ALL cells can perform a metabolic adaptation by promoting glutamine synthesis. By contrast, Notch‐positive T‐ALL cells maintain glutamine catabolism during glutamine restriction, leading to glutamine addiction and mTORC1 dependency.

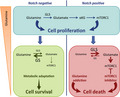

**Abbreviations**
7‐AAD: 7‐Aminoactinomycin D
BPTES: bis‐2‐(5‐phenylacetamido‐1,2,4‐thiadiazol‐2‐yl)ethyl sulfide
DON: diazo‐5‐oxo‐L‐norleucine
ECAR: extracellular acidification rate
GDH: glutamate dehydrogenase
GLS: glutaminase
GS: glutamine synthetase
GSI: γ‐secretase inhibitor
MSO: L‐methionine sulfoximine
mTORC1: mammalian target of rapamycin complex 1
NICD: Notch intracellular domain
PI: propidium iodide
RAP: rapamycin
T‐ALL: T‐cell acute lymphoblastic leukemia
TCA: tricarboxylic acid
αKG: α‐ketoglutarate

## 1 Introduction

T‐cell acute lymphoblastic leukemia (T‐ALL) appears upon the malignant transformation of a T‐cell progenitor. T‐ALL is frequently driven by the oncogenic receptor Notch1. However, treatments targeting Notch signaling result in resistant or relapsed disease, and still 20% of childhood patients and 40% of adult patients do not survive [1]. Thus, a better understanding of the molecular basis of T‐ALL origin and progression is essential for the proposal, design, and validation of more specific, highly effective treatments against this type of leukemia.

Notch receptors (Notch1‐4) are heterodimeric peptides, including an extracellular subunit and a transmembrane and intracellular subunit which interact through a heterodimerization domain present in both subunits. When a ligand of the DSL (Delta/Serrate/lag‐2) family located in the surface of a neighbor cell binds to the extracellular domain of the Notch receptor, it induces sequential cleavages in Notch by an ADAM metalloprotease and by a γ‐secretase, releasing the Notch intracellular domain (NICD) from the membrane [2]. NCID then translocates to the nucleus, interacts with specific DNA‐binding proteins (CBF1/Suppressor of Hairless/LAG‐1 and Mastermind/SEL‐8), and activates the transcription of target genes, such as the two families of transcriptional factors HES and HEY (including HES1, HES5, HEY1, and HEY2). The analysis of Notch1‐target genes and gene expression programs controlled by Notch1 showed that Notch1 promotes leukemic cell growth via direct transcriptional upregulation of genes involved in ribosome biosynthesis, amino acid metabolism, protein translation, and nucleotide synthesis. However, Notch1 activation also follows an indirect mechanism to induce leukemic transformation through the upregulation of key target pathways, namely c‐MYC pathway, PI3K/AKT pathway, and interleukin 7 receptor alpha chain. In addition, Notch1 activation increases G1/S cell cycle progression in T‐ALL through the upregulation of CCND3, CDK4, and CDK6 cell cycle genes [3].

Although seminal works already showed the importance of glutamine in the control of metabolism of human leukemia long ago [4, 5], still today we do not understand the role of glutamine metabolism in leukemia progression. Glutamine has been described as a crucial nutrient for many types of tumor. This amino acid is metabolized within the mitochondria through an enzymatic process termed glutaminolysis, whereby glutamine is transformed into α‐ketoglutarate (αKG), an intermediate of the tricarboxylic acid (TCA) cycle. Glutaminolysis is catalyzed by the enzymes glutaminase (GLS) and glutamate dehydrogenase (GDH) [6]. In addition to sustaining metabolism, glutaminolysis can also induce cell signaling deregulation in cancer cells through the hyperactivation of the mammalian target of rapamycin complex 1 (mTORC1) pathway [7, 8]. Conversely, glutamine synthesis through glutamine synthetase (GS) expression has been shown to be critical for the adaptation of certain types of solid tumors to glutamine scarcity [9].

In the case of Notch1‐driven T‐ALL, very little has been described regarding the participation of glutamine in Notch1‐mediated T‐cell malignant transformation or even in other types of cancer. Further, the mechanistic relationship between Notch1 and glutamine in the control of cellular homeostasis is not clear, as contradictory conclusions have been obtained. For instance, an *in vivo* study using T‐ALL mouse models reported that glutaminolysis plays a critical role in leukemia progression downstream of Notch1. Glutaminolysis is thus proposed to be a key determinant of the response to anti‐Notch1 therapies, as the inhibition of Notch1 blocks glutaminolysis [10]. Confirming the control of GLS by Notch1, an independent study in glioblastoma cells reached similar conclusions, showing a decrease of intracellular glutamate after Notch1 blockade [11]. However, a comparative metabolomic study performed in myeloid leukemic cells reported that the upregulation of Notch1 signaling decreases the expression of GLS and GDH and decreases glutamine consumption [12]. This study also showed that an increase in glutamine utilization disrupts Notch signaling pathway, leading to a decrease in cleaved Notch1, in Notch activity, and in Hey1 expression.

Herein, we show that Notch1 activation induces glutamine addiction in T‐ALL cells both *in vitro* and *in vivo*. We observed that Notch1 upregulation leads to proteosomal degradation of GS, responsible for glutamine addiction in Notch1‐activated leukemic cells. Concomitantly, Notch1 also induces the upregulation of GLS and the subsequent activation of mTORC1 signaling pathway, leading to mTORC1 dependency in Notch‐driven T‐ALL. Beyond confirming the model by which Notch1 induces glutaminolysis, our results propose that Notch1 executes a program leading to the upregulation of glutamine catabolism and cell growth signaling, and blocking glutamine anabolism, which ultimately leads to glutamine addiction in Notch1‐driven leukemia. Our results also pointed at the potential therapeutic benefits of targeting glutamine availability and mTORC1 signaling specifically in Notch1‐positive T‐ALL patients.

## 2 Materials ad methods

### 2.1 Reagents and antibodies

Antibodies against Notch1 total (#3608, dilution 1 : 1000), cleaved Notch1 (#4147, dilution 1 : 1000), c‐myc (#13987, 1 : 1000), β‐actin (#4967, dilution 1 : 1000), cleaved caspase 3 (#9664, dilution 1 : 1000), cleaved caspase 8 (#, 1 : 1000), cleaved PARP (#5625, dilution 1 : 1000), S6 (#2217, dilution 1 : 1000), phospho‐S6 (Ser235/236) (#4856, dilution 1 : 1000), S6K (#2708, dilution 1 : 1000), and phospho‐S6K(T389) (#9205, dilution 1 : 1000) were obtained from Cell Signaling Technology (Danvers, MA, USA). Antibody against GS (#610517, dilution 1 : 1000) was obtained from BD Biosciences (San Jose, CA, USA). Antibody against GLS (ab93434, dilution 1 : 1000) was purchased from Abcam (Cambridge, UK). Antibody against hes1 (sc‐165996, dilution 1 : 1000) is from Santa Cruz Biotechnology (Dallas, Texas, USA). The secondary antibodies anti‐mouse (#7076, dilution 1 : 1000) and anti‐rabbit (#7074, dilution 1 : 1000) were obtained from Cell Signaling Technology. The mTORC1 inhibitors rapamycin (RAP) and temsirolimus as well as the GS inhibitor l‐methionine sulfoximine (MSO), z‐VAD‐FMK caspase inhibitor, bis‐2‐(5‐phenylacetamido‐1,2,4‐thiadiazol‐2‐yl)ethyl sulfide (BPTES),CB‐839, and diazo‐5‐oxo‐l‐norleucine (DON) GLS inhibitors were obtained from Sigma‐Aldrich (St. Louis, MO, USA). DAPT (γ‐secretase inhibitor, GSI) and MG132 (proteasome inhibitor) were purchased from Santa Cruz. SMARTvector lentiviral human GS shRNA were obtained from Dharmacon (Lafayette, CO, USA). The plasmid GS human tagged ORF clone was purchased from Origene (Rockville, MD, USA) and the empty vector pJS27 MND‐DEST SV40‐Blasticidin is a gift from R. Iggo (Institute Bergonié). The plasmid MND‐LUC‐IRES was obtained from the Vector Platform at the University of Bordeaux (France).

### 2.2 Cell lines and culture conditions

CUTLL1 and H33HJ‐JA1 (CRL8163) were obtained from the collection of the laboratory of Marisa Toribio (Spain). HPB‐ALL, LOUCY, MOLT4 were purchased from DMSZ. All the cells lines were grown in RPMI high glucose (4.5 g·L^−1^) (GIBCO, Waltham, MA, USA) supplemented with 10% of FBS (Dominique Dutscher, Bernolsheim, France), glutamine (2 mm), penicillin (100 U·mL^−1^; Sigma), and streptomycin (100 mg·mL^−1^; Sigma), at 37 °C, 5% CO_2_ in humidified atmosphere. Mycoplasma contamination check was carried out using the PCR Mycoplasma Test Kit (PromoCell, Heidelberg, Germany). For glutamine withdrawal experiments, cells are incubated in RPMI without glutamine (GIBCO) with dialyzed serum (Dutscher) during indicated time. Glutamine was added at 2 mm final concentration. The different inhibitors were used as follows: BPTES (30 µm), DON (30 µm), CB‐839 (10 µm), zVAD (20 µm), DAPT (10 μm), MG132 (5 µm), MSO (1 mm), RAP (100 nm).

### 2.3 Targeted UPLC‐MS metabolomics

Cells were centrifuged at 300 ***g***, 5 min and frozen in liquid nitrogen and stored at −80 °C. Cell pellets were lysed in 500 μL of a mixture of ice‐cold water/methanol/acetic acid with a tissue homogenizer (Precellys) at 3500 ***g*** for 20 s. Subsequently, 400 μL of the homogenate was transferred to a fresh tube and shaken at 200 ***g*** for 30 min at 4 °C. Next, the aliquots were centrifuged for 15 min at 18 000 ***g*** at 4 °C. Seventy‐five microliter of the supernatant was transferred to a fresh tube and placed at −80 °C for 20 min. The chilled supernatants were evaporated for 2 h. The resulting pellets were suspended in 100 μL water/acetonitrile/formic acid. Concentrations of all metabolites were determined with a semi‐quantitative method, using calibration curves with chemical standards. Samples were measured with an ultra‐high‐performance liquid chromatography (UPLC) system (Acquity; Waters, Manchester, UK) coupled to a time‐of‐flight mass spectrometer (ToF MS, SYNAPT G2; Waters). A 2.1 x 100 mm, 1.7 µm BEH amide column (Waters), thermostated at 40 °C, was used to separate the analytes before entering the MS. Solvent A (aqueous phase) consisted of 99.5% water, 0.5% formic acid, and 20 mm ammonium formate while solvent B (organic phase) consisted of 29.5% water, 70% MeCN, 0.5% formic acid, and 1 mm ammonium formate. In order to obtain separation of the analytes, the following gradient was used: from 5% A to 50% A in 2.4 min in curved gradient (as defined by Waters), from 50% A to 99.9% A in 0.2 min constant at 99.9% A for 1.2 min, back to 5% A in 0.2 min. The flow rate was 0.250 mL·min^−1^, and the injection volume was 2 μL. All samples were injected randomly. After every 16 injections, a quality control sample was injected. The MS was operated in either positive or negative electrospray ionization (ESI+, ESI−). Moreover, two different scan modes were used namely full scan (50–1200 Da) and enhanced duty cycle (EDC, optimized per metabolite). For all MS modes, the cone voltage was between 20 and 25 depending on the analyte. Capillary voltage in ESI+ was 250 V and in ESI− 500 V. A 2 ng·mL^−1^ leucine‐enkephalin solution in water/acetonitrile/formic acid (49.9/50/0.1 %v/v/v) was infused at 10 μL·min^−1^ and used for a lock mass which was measured each 36 s for 0.5 s. Spectral peaks were automatically corrected for deviations in the lock mass. Data for the following set of metabolites were obtained from full scan in either ESI+ or ESI− (indicated by the sign for the *m/z* value). Extracted ion traces were obtained for threonine (*m/z* = +120.0661), serine (*m/z* = +106.0504), choline (*m/z* = +104.107), succinic acid (*m/z* = −117.0188), fumaric acid (*m/z* 115.0031), pyruvic acid (*m/z* = −87.0082), malic acid (*m/z* = −133.0137), citric acid (*m/z* = −191.0192), and oxaloacetic acid (+ 2Na, *m/z* = −191.0206) in a 20 mDa window and subsequently smoothed (2 points, two iterations) and integrated with quanlynx software (Waters). Data for the following set of metabolites were obtained in EDC mode. EDC functions for MTA, glutamate, glutamine, methionine, SAMe, SAH, spermine, spermidine, and 2‐ketoglutaric acid were optimized for the mass of the analyte in question. The following metabolites were measured in ESI+ mode where MTA was measured in scan function 1 (EDC at 298), methionine in scan function 2 (EDC at 152), glutamate in scan function 3 (EDC at 148), glutamine in scan function 4 (EDC at 147), SAH in scan function 5 (EDC at 385), SAMe in scan function 6 (EDC at 399), spermidine in scan function 7 (EDC at 146), and spermine in scan function 8 (EDC at 203). 2‐Ketoglutaric acid was measured in ESI− in scan function 1 (EDC at 145). Extracted ion traces were obtained for MTA (*m/z* = +298.097), methionine (*m/z* = +150.0589), SAH (*m/z* = +385.1294), SAMe (*m/z* = +399.1451) , glutamate (*m/z* = +148.061), glutamine (*m/z* = +147.077), spermidine (*m/z* = +146.1657), spermine (*m/z* = +203.2236), and 2‐ketoglutaric acid (*m/z* = −145.0137), in a 20 mDa window and subsequently smoothed (2 points, two iterations) and integrated with quanlynx software (Waters). Concentrations in the samples were calculated with the power‐fitted calibration curves. Concentrations were converted into amount of analyte (pmol) per million of cells, taking in account the analyte loss due to sample workup. All samples were injected randomly and in duplicate. Each measurement represents average of three independent experiments, each experiment performed in triplicates. Metabolite concentration was expressed as pmol per million of cells. Alternatively, glutamate levels were determined using a chromatography coupled to a QTRAP® 6500+ LC‐MS/MS System (from SCIEX, Framingham, MA, USA), with a LC column (100 × 2.1 mm ID, particle size 2.6 µm) and a corresponding guard column. MRM data were processed using multiquant™ 3.0.2 Software (SCIEX).

### 2.4 Glycolytic capacity measurement

CUTLL1 and H33HJ‐JA1 cells were incubated in the presence or absence of glutamine for 72 h. To measure the glycolytic capacity, 4 × 10^6^ cells were seeded in Seahorse® WF RPMI medium in XFe24 cell culture microplate pretreated with poly‐d‐lysine and centrifuge 5 min at 300 ***g***. To eliminate residues of carbonic acid from the medium, cells were incubated for at least 30 min at 37 °C with atmospheric CO_2_ in a nonhumidified incubator. Extracellular acidification rate (ECAR) was assayed in a Seahorse® XF‐24 extracellular flux analyzer by the addition via port A of 100 mm of glucose (final concentration 10 mm), port B of 10 µm of oligomycin (final concentration 1 µm) and port C of 500 mm of 2‐DG (final concentration 50 mm). Three measurement cycles of 2‐min mix, 2‐min wait, and 4‐min measure were carried out at basal condition and after each injection. At the end of the experiment, each well was washed twice with 50 µL of PBS and proteins were extracted with 50 µL of RIPA lysis buffer at room temperature. Protein concentration in each well was measured by a bicinchoninic acid assay according to the manufacturer's instructions (Thermo Fisher, Waltham, MA, USA). ECAR was normalized based on protein quantification. Glycolytic capacity represents the maximum ECAR rate reached by a cell following the addition of oligomycin, effectively shutting down oxidative phosphorylation and driving the cell to use glycolysis to its maximum capacity.

### 2.5 Plasmids and transfections

The lentiviral production was carried out as described [13]. Subsequently, cells were seeded at a density of 500 000 cells per well in a 24‐well plate, infected using concentrated lentiviral supernatants at MOI5 for 24 h. Then, the cells were amplified and sorted by BD FACSAria sorting flow cytometer for GFP expression.

### 2.6 Western blot analysis

All cell lines were seeded in 10‐cm plates. After the treatment, cells were centrifuged at 300 ***g***, 5 min then washed with phosphate‐buffered saline (PBS 1×), and lysed on ice using RIPA buffer, supplemented with protease inhibitors (Sigma), phosphatase inhibitors (Sigma), and PMSF 1 mm (AppliChem, Darmstadt, Germany). Protein quantification was performed with bicinchoninic acid assay kit (Thermo Fisher). After the electrophoresis, the proteins were transferred to a nitrocellulose membrane (Bio‐Rad, Hercules, CA, USA) with Trans‐Blot Turbo Transfer System (Bio‐Rad). The membranes were incubated for 30 min in PBS 1× with 0.01% Tween‐20 and 5% BSA. Incubation with primary antibodies is overnight at 4 °C and incubation with secondary antibodies is 2 h at room temperature. Finally, membranes were imaged using the Chemi Doc MP Imager (Bio‐Rad).

### 2.7 Cell viability analysis

To assess cell proliferation, viability, and death, cells were seeded in 125 000 cells per mL in 24‐well plates for 7 days counting in triplicate. The number of total and alive cells was determined using the TC20 Automated Cell Counter (Bio‐Rad) according to the manufacturer's instructions. Cells were counted every day with the counter with trypan blue 5% solution (Bio‐Rad). After 7 days, proliferation curve has been established with the standard derivative. Cell viability was then calculated as an end‐point or for 7 days in triplicate.

### 2.8 Flow cytometry analysis

For apoptotic cell death, after treatment, cells were stained with annexin V and propidium iodide (PI) or 7‐aminoactinomycin D (7‐AAD) following the manufacturer's protocol (BD Biosciences). Then, annexin V and PI/7‐AAD staining were analyzed using BD FACS Canto BD Biosciences flow cytometer. The analysis of the data was performed using the software facs diva (BD Biosciences).

### 2.9 Real‐time PCR

mRNA extraction was performed with Trizol (Invitrogen, Carlsbad, CA, USA). One microgram of total mRNA was reverse‐transcribed using the GoScript Reverse Transcription system (Promega, Madison, WI, USA) following the manufacturer's protocol. Quantitative real‐time PCR was performed using SSO Advanced Universal SYBR Green Supermix (Bio‐Rad). Expression levels of each gene were evaluated with normalization to RPL29 and GAPDH housekeeping genes. The sequence of the specific primers used for rtPCR is listed in the following table:

**Table:** 

Gene	Forward primer	Reverse primer
Notch1	AACAGCGAGGAAGAGGAGGA	GCATCAGAGCGTGAGTAGCG
NICD	AGTCCTCC GACAGACTGAGT	TCTTCTTGCTGGCCTCAGAC
Hes1	AGGCTGGAGAGGCGGCTAAG	TGGAAGGTGACACTGCGTTGG
Hey1	TGAGCTGAGAAGGCTGGTACCCA	TGCGCGTCAAAGTAACCTTTCCC
c‐myc	CTTCTCTCCGTCCTCGGATTCT	GAAGGTGATCCAGACTCTGACCTT
GS	TCATCTTGCATCGTGTGTGTG	CTTCAGACCATTCTCCTCCCG
GLS	TGGTGGCCTCAGGTGAAAAT	CCAAGCTAGGTAACAGACCCTGTT
GAPDH	CCATCTTCCAGGAGCGAGATC	GCCTTCTCCATGGTGGTGAA
RPL29	GGCTATCAAGGCCCTCGTAAA	CGAGCTTGCGGCTGACA

### 2.10 Glutamine uptake analysis

Glutamine uptake was assessed with ^3^H‐labeled glutamine. Cells were seeded at the concentration of 500 000 cells per mL in RPMI without glutamine for 4 h. Labeled glutamine was added at the concentration of 2.5 µCi and incubated for 15 min. Cells were collected and centrifuged at 1000 ***g*** in 4 °C for 5 min. Cell lysis was done after two washes in cold PBS with lysis solution (0.2N NaOH, 0.2% SDS) followed by HCl 2N. Protein concentration was quantified then the radioactivity was quantified in 5 mL of scintillation solution. We obtained the values that are normalized to protein content.

### 2.11 Animal models

All animals are maintained in the Animal Facility A2 of the University of Bordeaux (institutional agreement number: A33063916), led by B. Rousseau. The project has received the agreement of the Ethic Committee under the number APAFiS #94212017032614365349v7. We used 8‐week‐old male NOD.Cg‐*Prkdc^scid^ Il2rg^tm1Wjl^*/SzJ immunodeficient mice which were randomly assigned to the different treatment groups (20 mice/group). In the condition of glutamine withdrawal, the mice received glutamine‐free diet from 5‐week‐old and at 8‐week‐old mice received retro‐orbital injections of 0.5 × 10^6^ EV or NICD H33HJ‐JA1 cells which are luciferase positive. Mice fed with a glutamine‐free diet did not show any morphological or behavioral difference with respect to their complete diet‐fed littermates during the duration of the whole treatment (4–5 weeks). We performed the first imaging 1 week after the injection and twice per week. We evaluated disease progression and therapy response by *in vivo* luminescence bioimaging with the PhotonIMAGER (Biospace Lab, Nesles la Vallée, France). Temsirolimus (4 mg·kg^−1^) was injected intraperitoneally twice a week (every 3–4 days). When mice were sacrificed after 4 weeks of injection, blood and bone marrow samples were collected and analyzed for further analyses. Glutamine concentrations were determined in mice sera using the YSI 2950 Biochemistry Analyzer (YSI Life Sciences, Yellow Springs, OH, USA). Human CD3‐positive cells were analyzed by FACS using PE‐coupled anti‐human CD3 antibody (BD Biosciences).

### 2.12 Statistical analysis

The results are expressed as a mean ± SEM of at least three independent experiments. One‐way or two‐way ANOVA followed by Bonferroni's comparison as a *post hoc* test was used to evaluate the statistical difference between more than two groups. *t*‐Test analysis was used to evaluate the statistical difference between two groups. Statistical significance was estimated when *P* < 0.05.

## 3 Results

### 3.1 Glutamine sustains TCA cycle in T‐ALL cells

**Fig. 1:**
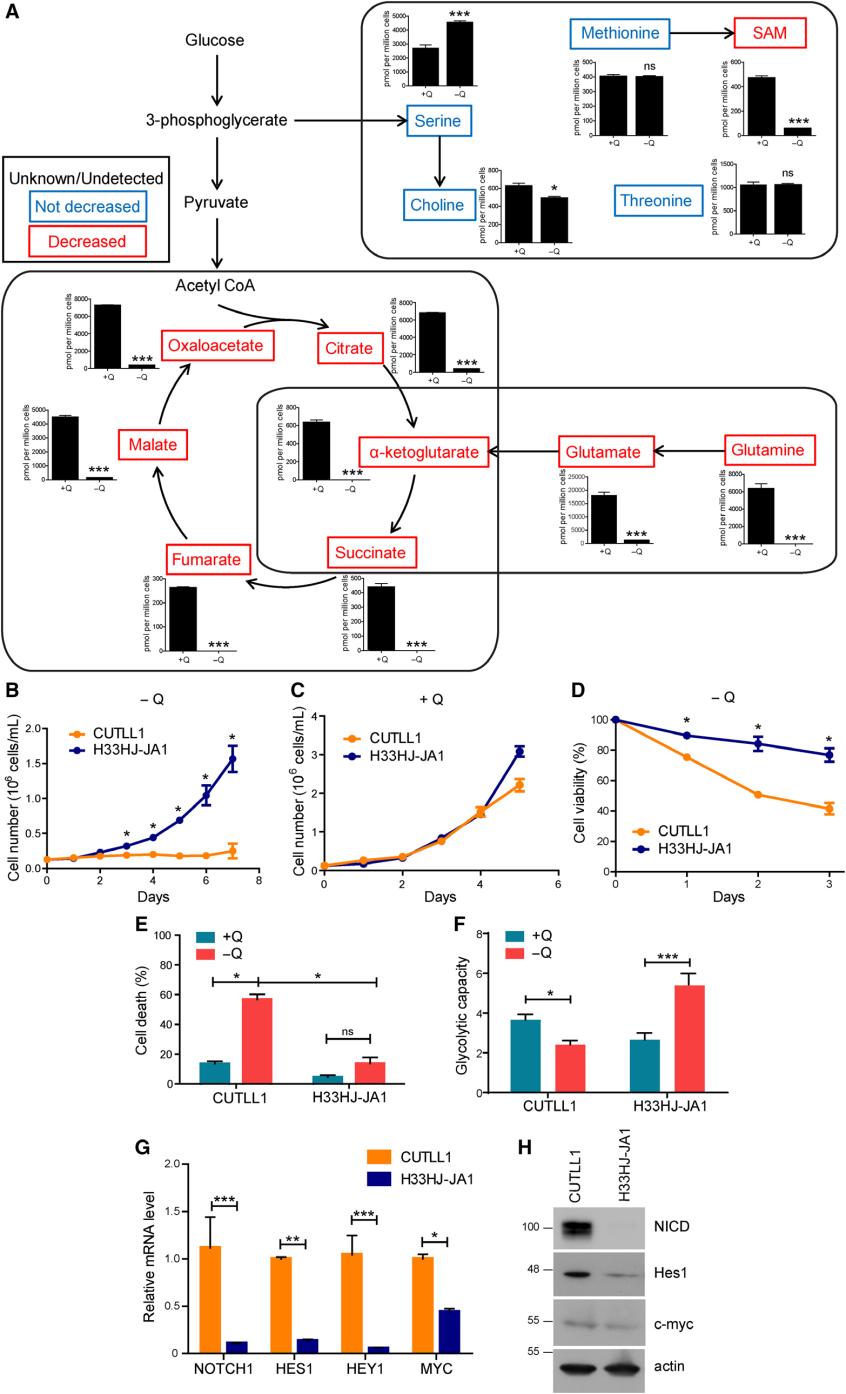
Glutamine sustains TCA cycle in T‐ALL cells. (A) Targeted metabolomic analysis of H33HJ‐JA1 cells incubated either in the presence (+Q) or the absence (−Q) of glutamine for 24 h. The concentration of metabolites belonging to glutaminolysis, TCA cycle, or glycolysis was analyzed by mass spectrometry. Unchanged metabolites are highlighted in blue; metabolites which concentration decreased in the absence of glutamine are highlighted in red. Graphs show mean values ± SEM (*n* = 3). (B‐C) Growing curves of CUTLL1 and H33HJ‐JA1 cells incubated in the absence (B) or presence (C) of glutamine for the indicated times. Cell concentration was determined using a cell counter. (D) CUTLL1 and H33HJ‐JA1 cells were incubated in the absence of glutamine for the indicated times. Then, cell viability was estimated using a trypan blue assay. (E) Cell death of CUTLL1 and H33HJ‐JA1 cells incubated either in the presence (+Q) or absence (−Q) of glutamine during 72 h. Cell death was estimated using a trypan blue assay. (F) Glycolytic capacity of CUTLL1 and H33HJ‐JA1 cells incubated in the presence (+Q) or absence (−Q) of glutamine during 72 h. Glycolytic capacity was determined using Seahorse® technology as described in Methods section. (G) Relative mRNA levels of NOTCH1, HES1, HEY1, and MYC in CUTLL1 and H33HJ‐JA1 cells incubated in the presence of glutamine. (H) Western blot analysis of the protein levels of NICD, Hes1, c‐myc, and actin, in CUTLL1 and H33HJ‐JA1 cells incubated in the presence of glutamine. Graphs show mean values ± SEM (*n* ≥ 3, *P < 0.05; **P < 0.01; ***P < 0.001). Two‐way ANOVA followed by Bonferroni's comparison as a *post hoc* test was used to evaluate the statistical difference between more than two groups. *t*‐Test analysis was used to evaluate the statistical difference between two groups.

To better understand the role of glutamine in the metabolism of T‐ALL, we performed a metabolomic analysis in two different T‐ALL cell types (H33HJ‐JA1 and CUTLL1) in response to glutamine deprivation (Fig. S1a‐b). As expected, the levels of glutamine, glutamate, and αKG were decreased in both cell lines when cells were incubated in the absence of glutamine (Fig. 1A), confirming that glutaminolysis was abrogated in cells incubated in the absence of glutamine. However, we also observed a profound decrease in the levels of TCA cycle components such as succinate, malate, fumarate, oxaloacetate, and citrate (Fig. 1A), sustaining a key role of glutamine in the support of TCA cycle through glutaminolysis in T‐ALL cells. In contrast, the levels of other metabolites not sustained by the TCA cycle (such as threonine, serine, choline, and methionine) were not decreased (Fig. 1A). These results highlighted the specificity of glutamine in sustaining the TCA cycle in lymphoblastic leukemia and led us to further investigate the addiction of T‐ALL cells to glutamine availability.

### 3.2 Notch1 activation correlates with glutamine addiction in T‐ALL cells

**Fig. 2:**
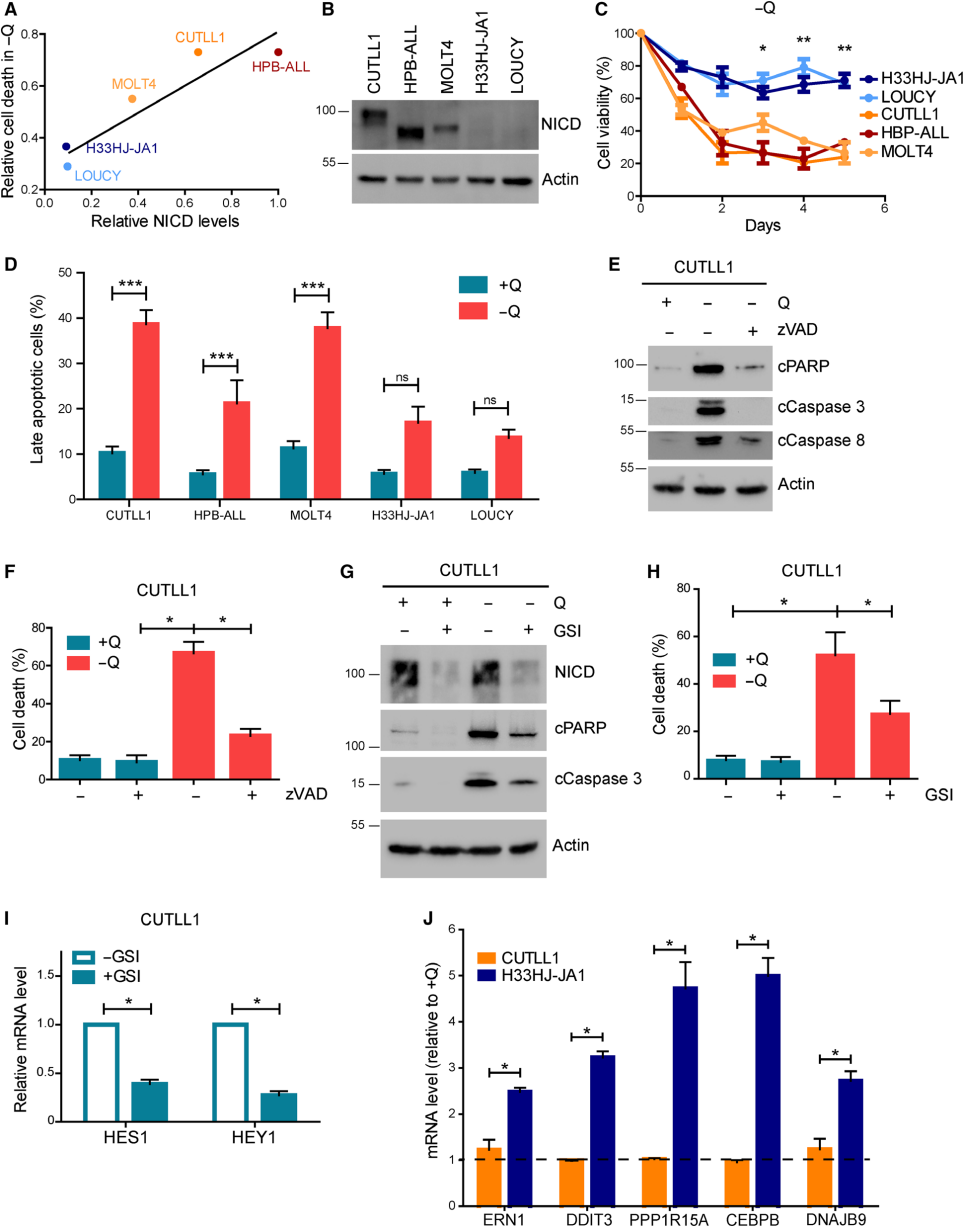
Notch1 activation correlated with glutamine addiction in T‐ALL cells. (A) The relative levels of NICD and cell death induction during glutamine restriction for 72 h were estimated for five different T‐ALL cell lines. Values were represented in the graph, and the linear regression was calculated and represented. (B) CUTLL1, HBP‐ALL, MOLT4, H33HJ‐JA1, and LOUCY cells were incubated in complete medium for 24 h. Cell extracts were collected and levels of NICD and actin were estimated by western blot. (C) CUTLL1, HBP‐ALL, MOLT4, H33HJ‐JA1, and LOUCY cells were incubated in the absence of glutamine for the indicated times and cell viability was determined using a trypan blue assay. (D) CUTLL1, HBP‐ALL, MOLT4, H33HJ‐JA1, and LOUCY cells were incubated either in the presence or the absence of glutamine (Q) during 72 h as indicated. Then, late apoptotic cell percentage was quantified through flow cytometry analysis of PI and annexin V content. (E‐F) CUTLL1 cells were incubated either in the presence or absence of glutamine (Q) and zVAD (20 µm) during 72 h as indicated. Cell extracts were collected and levels of cleaved PARP, cleaved caspase 3, cleaved caspase 8, and actin were estimated by western blot (E), while cell death was estimated using a trypan blue assay (F). (G) Western blot analysis of the apoptotic markers cleaved PARP and cleaved caspase 3 of CUTLL1 cells incubated either in the presence or the absence of glutamine (Q) and GSI (DAPT 10 µm) during 72 h as indicated. (H) Percentage of dead cells, as estimated using a trypan blue assay, of CUTLL1 cells incubated as in G. (I) CUTLL1 cells were incubated in the presence or absence of GSI (DAPT 10 µm) during 72 h in complete medium as indicated. RNA content of cells was extracted and HES1 and HEY1 mRNA level was estimated by quantitative PCR. (J) CUTLL1 and H33HJ‐JA1 cells were incubated either in the presence or absence of glutamine for 72 h as indicated. RNA content of cells was extracted, and RNA levels of ERN1, DDIT3, PPP1R15A, CEBPB, and DNAJB9 were estimated by quantitative PCR. Values represent RNA content of each gene of cells incubated in the absence of glutamine with respect to the RNA content of the same gene of cells incubated in the presence of glutamine. Graphs show mean values ± SEM (*n* ≥ 3, *P < 0.05). Two‐way ANOVA followed by Bonferroni's comparison as a *post hoc* test was used to evaluate the statistical difference between more than two groups.

Next, we investigated glutamine addiction in T‐ALL cells. Surprisingly, and despite the metabolic importance of glutamine in both T‐ALL cells, only H33HJ‐JA1 cells, but not CUTLL1 cells, were able to proliferate in glutamine‐free conditions (Fig. 1B). No major differences were observed in glutamine‐rich conditions among these cells (Fig. 1C). The lack of cell proliferation of CUTLL1 cells incubated in the absence of glutamine correlated with a drastic increase in cell death, not observed in H33HJ‐JA1 cells (Fig. 1D,E). Correlating with their capacity to survive in glutamine‐deprived conditions, H33HJ‐JA1 also showed an increase in the glycolytic capacity upon glutamine withdrawal, not observed in CUTLL1 cells (Fig. 1F). Thus, upregulating glycolysis might constitute a mechanism of adaptation to glutamine scarcity in these cells. One of the main genetic differences between these two cell lines is their differential activation of Notch1 as observed by the expression levels of NICD and Notch1 downstream targets Hes1, Hey1, and c‐myc: basal activity in H33HJ‐JA1 cells (as previously described for these cells [14]), high activity in CUTLL1 (Fig. 1G,H). Hence, we investigated the correlation between Notch1 activation and glutamine addiction using a broader panel of T‐ALL cells. As shown in Fig. 2A, glutamine addiction positively correlated with Notch1 activity in T‐ALL cells, as determined by the levels of NICD and cell viability under glutamine restriction in a panel of T‐ALL cell lines (Fig. 2B,C). Indeed, glutamine restriction induced apoptosis in Notch1‐positive T‐ALL cells (CUTLL1, HPB‐ALL, and MOLT4), but not in Notch1‐negative cells (H33HJ‐JA1 and LOUCY), as determined by the population of annexin V/PI‐positive cells (Figs 2D and S2a). Confirming apoptosis induction in glutamine‐restricted Notch1‐positive cells, the treatment with zVAD, a caspase inhibitor, reduced PARP and caspase cleavage, and rescued cell viability (Fig. 2E,F) in these conditions. The specific, positive role of Notch1 signaling in glutamine addiction of T‐ALL cells was also sustained by the reduction of apoptotic cell death in glutamine‐deprived Notch‐positive T‐ALL cells upon GSI treatment, a Notch1 inhibitor (Fig. 2G,H). The efficient inhibition of Notch1 signaling by GSI was confirmed by the reduced levels of NICD and by the reduced expression of the Notch1 downstream target genes *hes1* and *hey1* (Fig. 2I). Intriguingly, the inability of Notch1‐positive T‐ALL cells to survive in glutamine‐free conditions correlated with their lack of induction of an appropriate UPR response in these conditions, a defect not seen in Notch1‐negative cells (Fig. 2J). Altogether, these results clearly suggest that Notch1 upregulation correlates with an induced addiction to glutamine in T‐ALL cells.

### 3.3 Notch1 upregulation induces glutamine addiction in T‐ALL cells

**Fig. 3:**
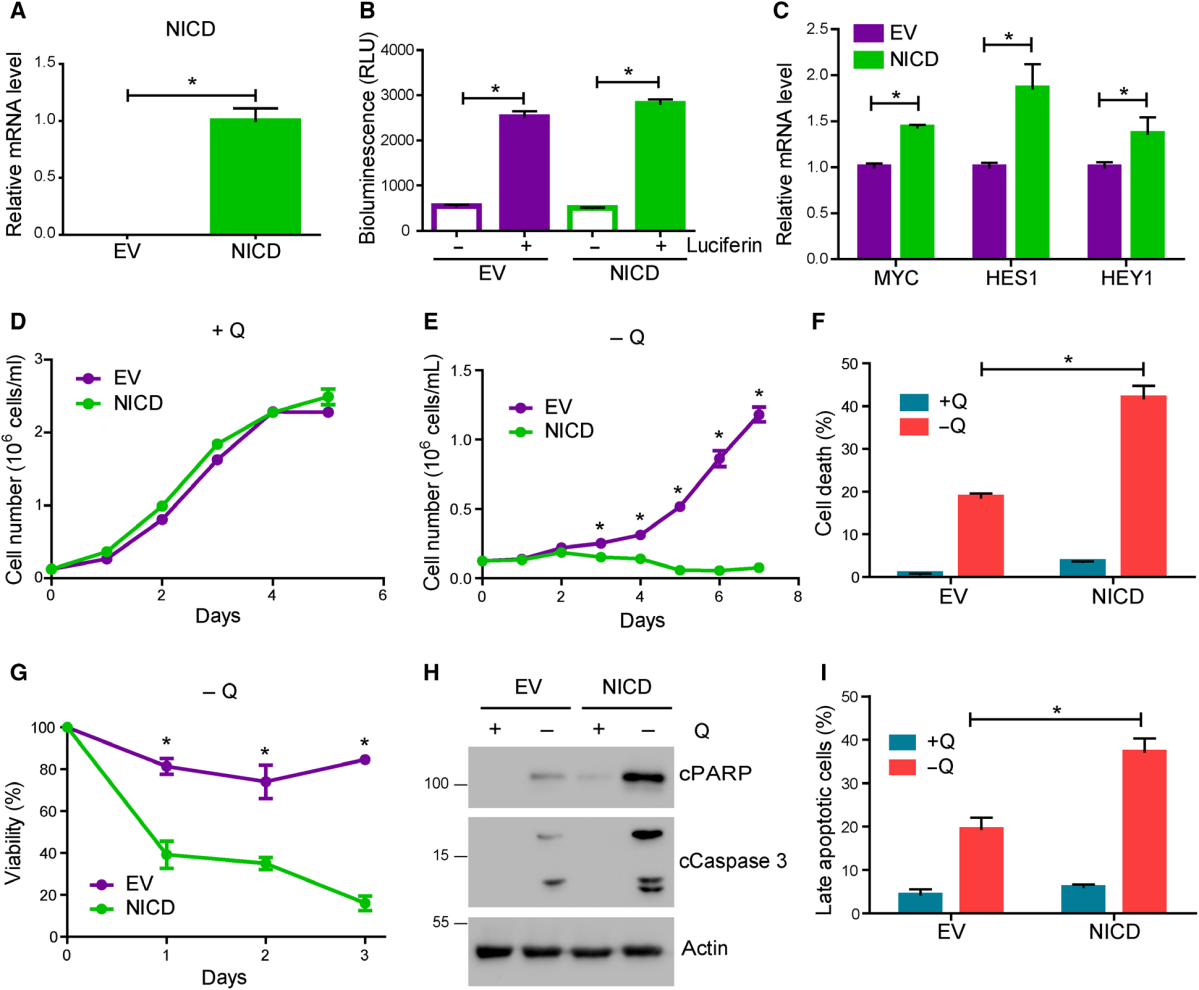
Notch1 upregulation induced glutamine addiction in T‐ALL cells. (A) RNA levels of NICD, as estimated by quantitative PCR, of EV and NICD H33HJ‐JA1 cells cultivated in complete medium. (B) Luciferase‐dependent luminescence was estimated in EV and NICD cells using a luminometer. (C) RNA content of EV and NICD cells was extracted from cells cultivated in complete medium. MYC, HES1, and HEY1 mRNA levels were estimated by quantitative PCR. (D‐E) Growing curves of EV and NICD cells incubated in the presence (D) or absence (E) of glutamine for the indicated times. Cell concentration was determined using a cell counter. (F) Cell death of EV and NICD cells incubated either in the presence (+Q) or absence (−Q) of glutamine during 72 h. Cell death was estimated using a trypan blue assay. (G) Cell viability, as estimated by trypan blue assay, of EV and NICD incubated in the absence of glutamine for the indicated time. (H) Western blot analysis of the apoptotic markers cleaved PARP and cleaved caspase 3 of EV and NICD cells incubated as in F. (I) Late apoptotic cell percentage, as quantified by flow cytometry analysis of PI and annexin V content, of EV and NICD cells incubated as in F. Graphs show mean values ± SEM (*n* ≥ 3, *P < 0.05). Two‐way ANOVA followed by Bonferroni's comparison as a *post hoc* test was used to evaluate the statistical difference between more than two groups. *t*‐Test analysis was used to evaluate the statistical difference between two groups.

The results shown above led us to investigate the sufficiency of Notch1 to induce glutamine addiction in T‐ALL cells. For this purpose, we stably infected H33HJ‐JA1 cells (Notch1‐negative cells) either with an empty vector (herein after referred as ‘EV cells’) or with a vector expressing NICD (‘NICD cells’). The correct expression of NICD in infected cells was analyzed by qPCR (Fig. 3A). To track them, both EV and NICD cells were co‐infected with a luciferase reporter (Fig. 3B). The efficient upregulation of Notch1 signaling in NICD cells was confirmed by the increased expression levels of the Notch1 downstream targets *c‐myc*, *hes1*, and *hey1* (Fig. 3C). The upregulation of NICD did not affect the capacity of these leukemic cells to proliferate in glutamine‐rich conditions (Fig. 3D). However, while EV cells, as their parental counterpart, were able to proliferate in the absence of glutamine, upregulation of Notch1 in NICD cells abolished dramatically their capacity to proliferate upon glutamine withdrawal (Fig. 3E). The lost capacity to proliferate in glutamine‐restricted conditions observed in NICD cells was accompanied by a dramatic increase in cell death, not observed in EV cells (Fig. 3F‐H). As previously observed for Notch1‐positive leukemic cells, apoptosis induction accounted for this increase in cell death. Thus, as shown in Fig. 3H, NICD cells showed a prominent increase in pro‐apoptotic markers, such as cleaved PARP and cleaved caspase 3, not observed in EV cells. Induction of apoptosis in NICD expressing cells upon glutamine withdrawal was again confirmed by annexin V / PI staining analysis by flow cytometry (Figs 3I and S2b). These results confirmed the sufficiency of Notch1 signaling to induce glutamine addiction in lymphoblastic leukemic cells.

### 3.4 Glutamine‐free diet impairs Notch1‐driven leukemia progression *in vivo*

**Fig. 4:**
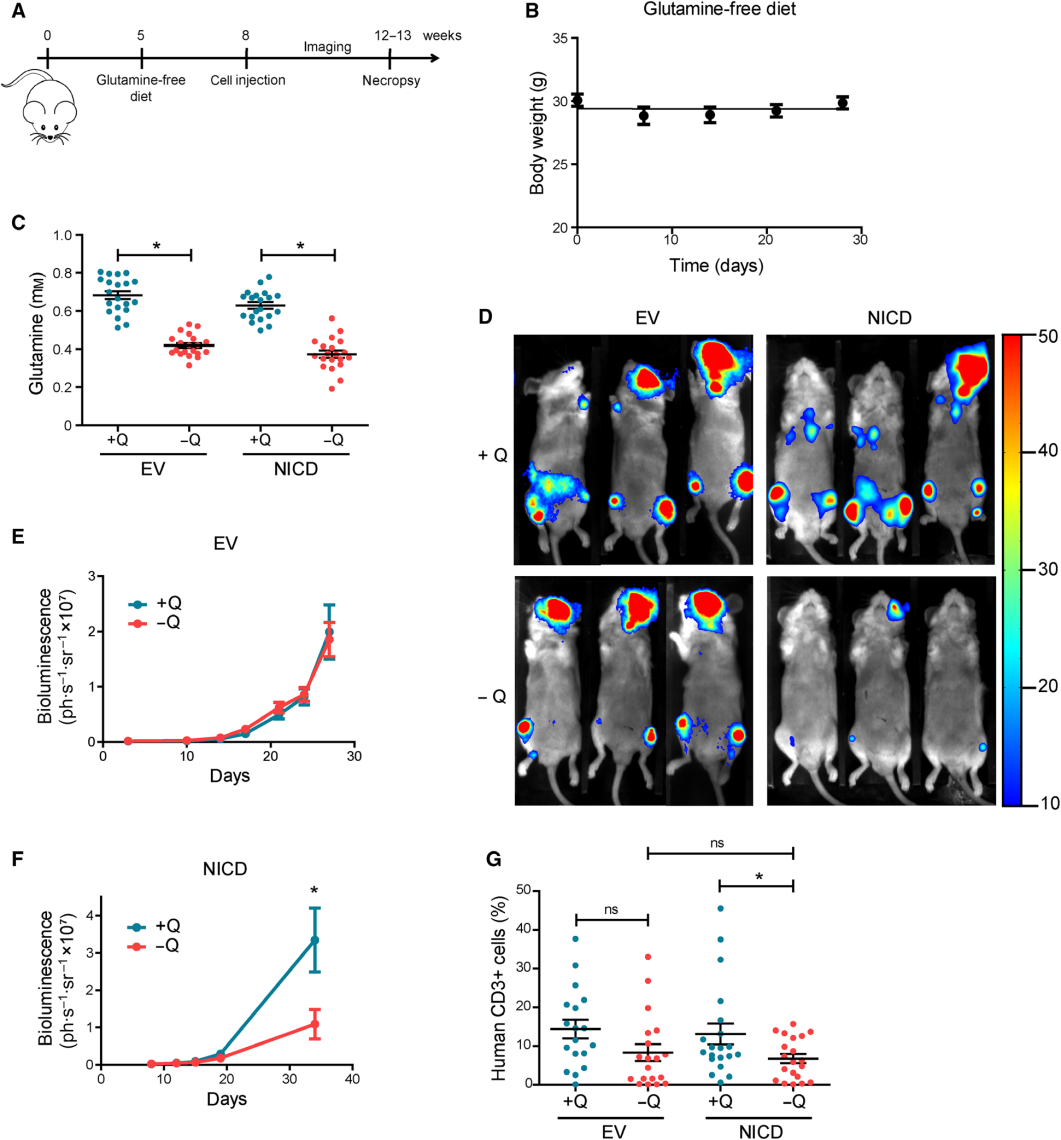
Glutamine‐free diet impairs Notch1‐driven leukemia progression *in vivo*. (A) Schematic representation of the strategy followed for *in vivo* experiments. (B) Evolution of body weight of mice fed with a glutamine‐free diet for the indicated time. (C) Concentration of glutamine in the blood of mice implanted with either EV or NICD H33HJ‐JA1 cells fed under complete (+Q) or glutamine‐free (−Q) diet at the end of the treatment, as indicated. (D‐F) Representative luminescence images (D) and luminescence quantification (E‐F) of mice implanted with either EV or NICD cells fed under complete (+Q) or glutamine‐free (−Q) diet at the end of the treatment, as indicated. (G) Infiltration of leukemic human (CD3+) cells in the bone marrow of mice implanted with either EV or NICD cells, fed either in the presence or the absence of glutamine (Q). Graphs show mean values ± SEM (20 mice/group, *P < 0.05). Two‐way ANOVA followed by Bonferroni’s comparison as a *post hoc* test was used to evaluate the statistical difference between more than two groups.

Next, validating the physiological relevance of our previous observations, we investigated if Notch1‐positive cells were addicted to glutamine *in vivo*. For this purpose, we injected both EV and NICD H33HJ‐JA1 cells (luciferase positive) into mice receiving either a normal complete diet or a diet in which both glutamine and glutamate content was eliminated (Fig. 4A). No significant body weight changes were observed in mice fed with either a complete or a glutamine‐free diet (Fig. 4B). Glutamine levels in the blood of mice fed with a glutamine‐free diet decreased significantly with respect to the levels of glutamine in the blood of mice fed with a glutamine‐rich diet, with no significant differences observed between mice injected with EV cells or NICD cells (Fig. 4C). Despite persistence of cells in the skulls due to the retro‐orbital injection site, NICD cells were able to induce leukemia progression (determined by *in vivo* luciferase analysis) in complete diet‐fed mice similarly to EV cells (Fig. 4D, top panels). However, disease progression induced by NICD leukemic cells was strongly impaired in glutamine‐free fed mice (Fig. 4D, bottom right panel). In contrast, EV cells (Noch1‐negative cells) were able to promote leukemia progression in both glutamine‐free and glutamine‐rich fed mice (Fig. 4D, left panels). Thus, a glutamine‐restricted diet prevented leukemia progression specifically in mice injected with Notch1‐positive leukemic cells (Fig. 4E,F). A necropsy analysis revealed a modest, yet significant reduction of NICD‐positive cells (assessed by CD3+ cells) infiltrated in the bone marrow of mice fed in the absence of glutamine with respect to mice fed in the presence of glutamine (Fig. 4G). This difference was not observed in NICD negative cells (EV cells). These results confirmed that Notch1‐positive T‐ALL cells are addicted to glutamine *in vivo*.

### 3.5 Notch1 modulates glutamine metabolizing enzymes in T‐ALL cells

**Fig. 5:**
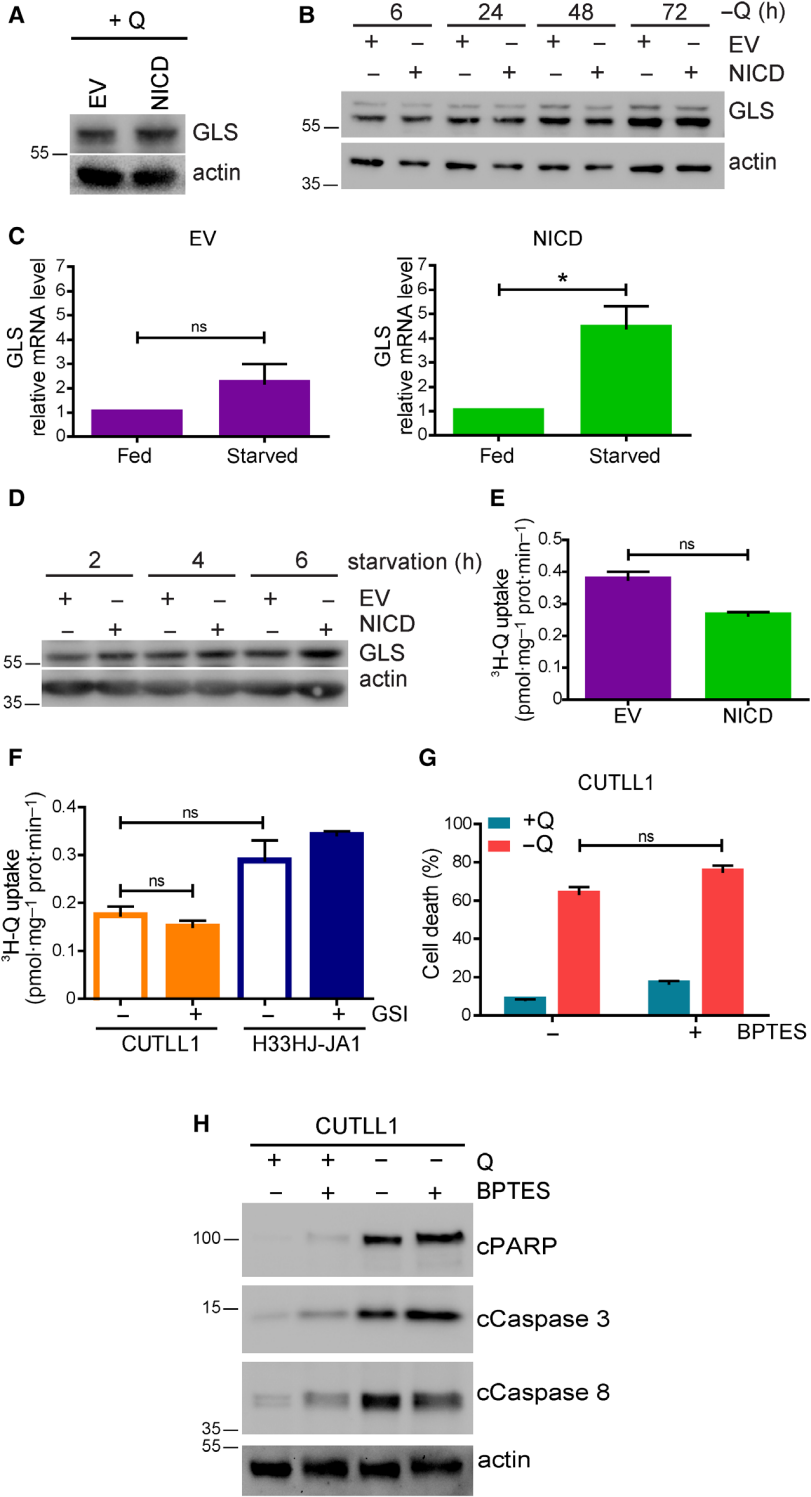
Notch1 modulated glutamine metabolizing enzymes in T‐ALL cells. (A) EV and NICD cells were incubated in a complete medium for 24 h. Cell extracts were collected and levels of GLS and actin were estimated by western blot. (B) EV and NICD cells were incubated in the absence of glutamine (−Q) for the indicated time. GLS and actin levels were determined by western blot analysis. (C) EV and NICD cells were incubated either in a complete medium (fed) or in a medium without amino acids (starved) during 72 h as indicated. Then, RNA content of these cells was extracted and GLS mRNA level was estimated by quantitative PCR. (D) EV and NICD cells were incubated in a medium without amino acids for the indicated time. Cell extracts were collected and levels of GLS and actin were estimated by western blot. (E) EV and NICD cells were incubated in the absence of glutamine and then incubated with radiolabeled ^3^H‐glutamine during 15 min. Cell content was extracted, and radiolabeled glutamine uptake was measured using a scintillation counter. (F) Glutamine‐starved CUTLL1 and H33HJ‐JA1 cells were incubated either in the presence or absence of GSI (DAPT 10 µm) for 72 h. Then, glutamine incorporation was determined as in E. (G‐H) CUTLL1 cells were incubated either in the presence or absence of glutamine (Q) and BPTES (30 µm) during 72 h as indicated. Cell death was estimated using a trypan blue assay (G), while cell extracts were collected and levels of cleaved PARP, cleaved caspase 3, cleaved caspase 8, and actin were estimated by western blot (H). Graphs show mean values ± SEM (*n* ≥ 3, *P < 0.05). Two‐way ANOVA followed by Bonferroni’s comparison as a *post hoc* test was used to evaluate the statistical difference between more than two groups. *t*‐Test analysis was used to evaluate the statistical difference between two groups.

In an attempt to understand the molecular mechanisms linking Notch1 and glutamine addiction, we investigated the effects of Notch1 induction on GLS levels [6]. Our results showed that the induction of Notch1 signaling in NICD cells did not affect the levels of GLS in nutrient‐rich conditions (Fig. 5A). While glutamine removal did not affect either GLS levels in Notch1‐positive T‐ALL cells (Fig. 5B), the levels of GLS (both at the RNA and protein level) were enhanced in NICD cells with respect to EV cells when these cells were incubated in all amino acid restricted conditions (Fig. 5C,D). This result led us to investigate the potential connection of Notch1 with glutamine catabolism. However, the observed upregulation of GLS by Notch1 did not correlate with an increase in the transport of glutamine in these cells (Fig. 5E). Accordingly, the pharmacological inhibition of Notch1 signaling using GSI did not affect significantly the uptake of glutamine (Fig. 5F). Thus, Notch1 seems to exert certain control on GLS expression, but does not regulate acute glutamine transport in leukemic cells. We reported recently that an increase in glutamine catabolism during nutritional imbalance induces a particular type of apoptotic cell death that we named ‘glutamoptosis’ [15, 16]. Thus, we investigated if glutamoptosis induction during glutamine restriction was the reason of Notch1‐induced glutamine addiction. Typically, glutamoptosis is inhibited by GLS inhibitors, such as BPTES, which reduces glutaminolysis [15]. However, the results shown in Fig. 5G,H indicated that BPTES treatment did not prevent the addiction to glutamine of Notch1‐driven leukemic cells, as no decrease neither in cell death induction nor in apoptotic markers was observed upon BPTES treatment. The efficiency of BPTES to inhibit GLS activity was confirmed by the observed reduced intracellular levels of glutamate upon BPTES treatment (Fig. S3a). Indeed, reduced glutamate intracellular levels were observed in cells treated with three different GLS inhibitors: BPTES, DON, and CB‐839. None of these inhibitors prevented cell death induced upon glutamine withdrawal in CUTLL1 cells (Fig. S3b). These results discarded glutamoptosis as a main reason for Notch1‐mediated glutamine addiction. In addition, it is worth noting that the inhibition of GLS using BPTES in Notch1‐positive cells during nutrient‐rich conditions did not induce the increase in apoptosis observed upon glutamine restriction. These results point at the very important conclusion that glutamine starvation and glutaminolysis inhibition render completely different responses in Notch1‐driven T‐ALL.

**Fig. 6:**
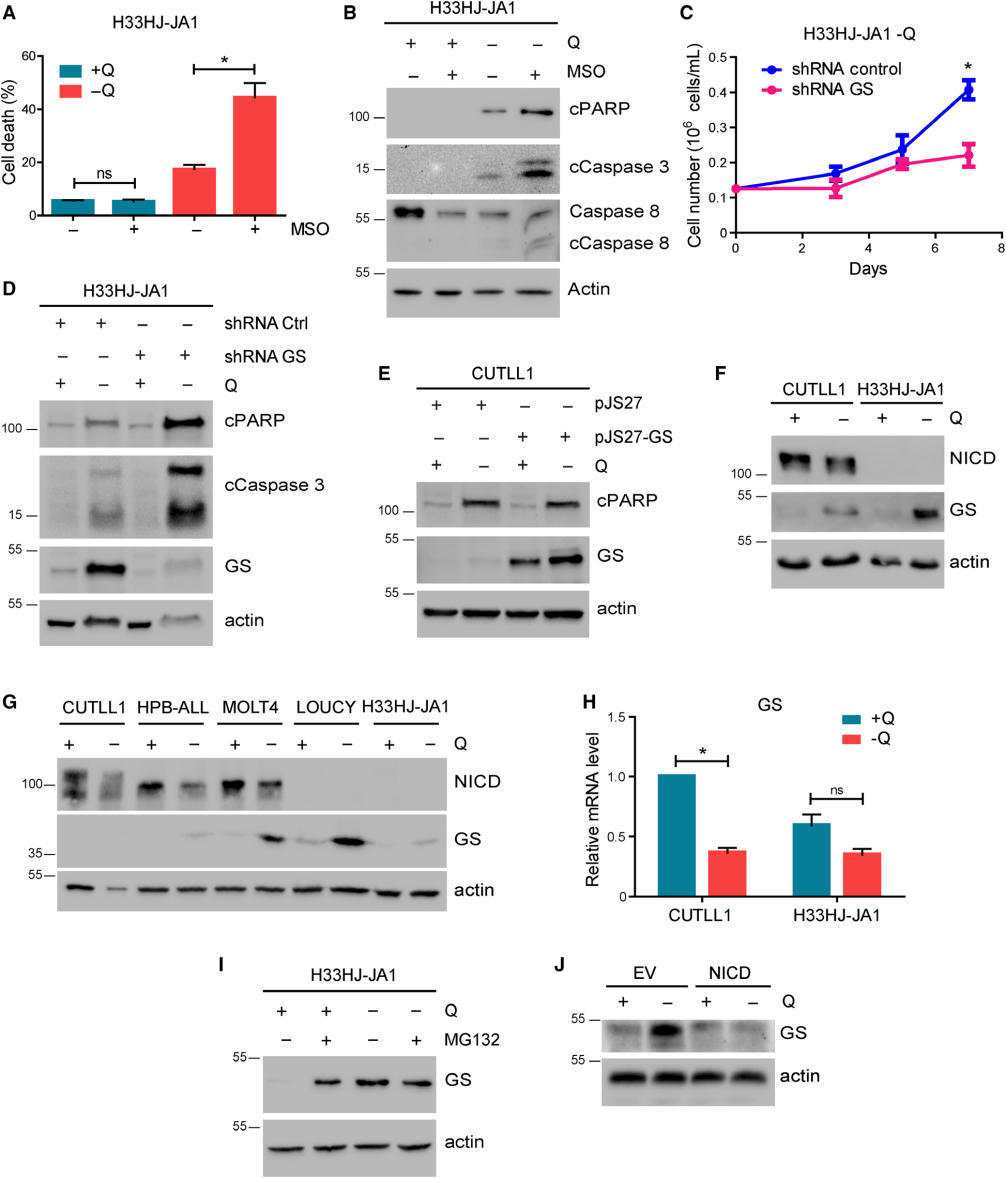
The lack of GS accumulation induced by Notch1 is responsible for glutamine addiction in Notch1‐positive T‐ALL cells. (A) Cell death, as estimated using a trypan blue assay, of H33HJ‐JA1 cells incubated either in the presence or the absence of glutamine (Q) and MSO (1 mm) during 72 h as indicated. (B) Western blot analysis of the apoptotic markers cleaved PARP, cleaved caspase 3, cleaved caspase 8, and actin of H33HJ‐JA1 cells incubated as in A. (C) Growing curves of H33HJ‐JA1 cells infected with either a plasmid expressing a control nontargeting shRNA (shRNA Control), or a plasmid expressing a shRNA against GS (shRNA GS), and incubated in the absence of glutamine for the indicated times. (D) Western blot analysis of cleaved PARP, cleaved caspase 3, GS and actin of H33HJ‐JA1 cells infected with either a plasmid expressing a control nontargeting shRNA (shRNA Control), or a plasmid expressing a shRNA against GS (shRNA GS), and incubated in the presence or absence of glutamine during 72 h as indicated. (E) Western blot analysis of cleaved PARP, GS and actin of CUTLL1 cells infected with either an empty vector plasmid (pJS27) or with a plasmid overexpressing GS (pJS27‐GS) and incubated either in the presence (+Q) or absence (−Q) of glutamine for 72 h. (F) Western blot analysis of NICD, GS and actin of CUTLL1 and H33HJ‐JA1 cells incubated either in the presence (+Q) or absence (−Q) of glutamine during 72 h as indicated. (G) CUTLL1, HBP‐ALL, MOLT4, H33HJ‐JA1, and LOUCY cells were incubated either in the presence or the absence of glutamine (Q) for 72 h. Cell extracts were collected and levels NICD, GS, and actin were estimated by western blot. (H) CUTLL1 and H33HJ‐JA1 cells were incubated either in the presence (+Q) or absence (−Q) of glutamine. RNA content was extracted and GS mRNA level was estimated by quantitative PCR. (I) H33HJ‐JA1 cells were incubated either in the presence or absence of glutamine (Q) and MG132 (5 µm) during 4 h as indicated. Cell extracts were collected and levels of GS and actin were estimated by western blot. (J) Western blot analysis of GS and actin of EV and NICD cells incubated either in the presence (+Q) or absence (−Q) of glutamine during 72 h as indicated. Graphs show mean values ± SEM (*n* ≥ 3, *P < 0.05). Two‐way ANOVA followed by Bonferroni's comparison as a *post hoc* test was used to evaluate the statistical difference between more than two groups.

In parallel, we also investigated if GS activity, the critical enzyme for glutamine biosynthesis, was necessary for the adaptation of T‐ALL cells to glutamine scarcity, as observed for other cancer types [9]. For this purpose, we inhibited GS activity pharmacologically using MSO. Our results showed that MSO treatment resulted in apoptotic cell death in H33HJ‐JA1 cells specifically in glutamine‐restrictive conditions, showing increased cell death and increased apoptotic markers in these conditions (Fig. 6A,B). These pharmacological results were confirmed by the genetic inhibition of GS using shRNA (Fig. 6C,D). Conversely, GS overexpression attenuated apoptotic marker induction of Notch1‐positive cells during glutamine restriction (Fig. 6E). Sustaining a role of GS in Notch1‐dependent glutamine addiction, we also observed that Notch1‐negative T‐ALL cells (H33HJ‐JA1) presented a significant induction of GS at the protein level during glutamine restriction, not observed in Notch1‐positive T‐ALL cells (CUTLL1) (Fig. 6D‐F). Although modest and partial, a negative correlation between Notch1 and GS induction was observed when we analyzed a broader panel of T‐ALL cell lines (Fig. 6G). However, no increase in GS, but rather a compensatory decrease at RNA level, was observed (Fig. 6H), suggesting a post‐transcriptional regulation of GS. A recent publication suggested that GS levels are downregulated by glutamine availability due to its proteasomal degradation [17]. Indeed, in T‐ALL cells, we confirmed that the inhibition of the proteasome using MG132 treatment induced GS levels during glutamine sufficiency (Fig. 6I), confirming that GS is expressed but subsequently degraded in conditions of glutamine availability. Further confirming the sufficiency of Notch1 signaling to prevent GS accumulation during glutamine restriction, we observed that NICD cells showed a substantial decrease in the levels of GS with respect to their counterpart EV cells upon glutamine withdrawal (Fig. 6J). These results confirmed that the lack of GS accumulation induced by Notch1 is responsible for glutamine addiction in Notch1‐positive T‐ALL cells.

### 3.6 mTORC1 inhibition synergizes with glutamine restriction to induce cell death and to prevent disease progression in Notch1‐positive T‐ALL

**Fig. 7:**
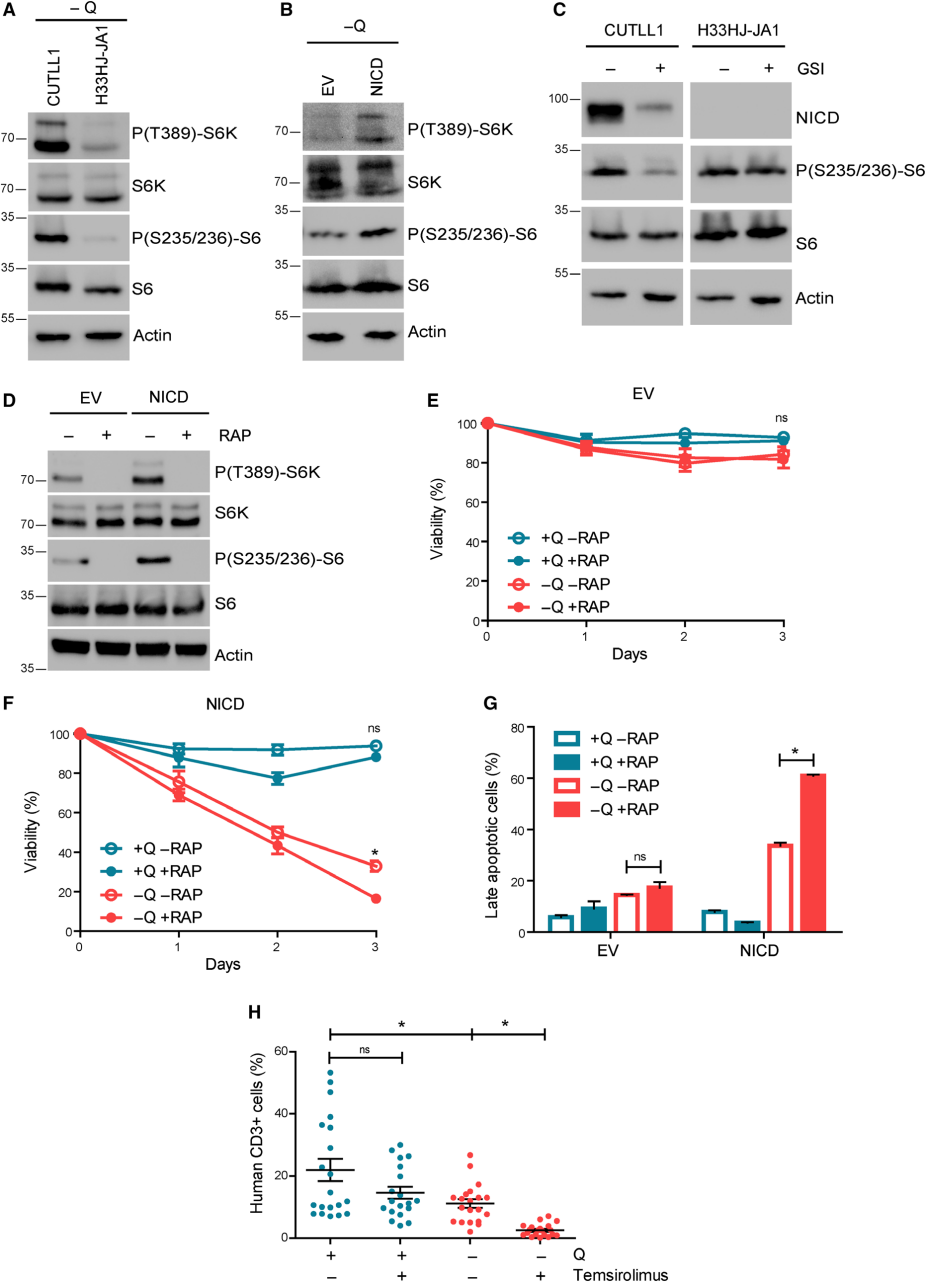
mTORC1 inhibition synergizes with glutamine starvation to induce cell death and to prevent leukemia progression in Notch1‐positive T‐ALL. (A) Western blot analysis of phospho‐S6K, total S6K, phospho‐S6, total S6, and actin of CUTLL1 and H33HJ‐JA1 cells incubated in the absence of glutamine during 72 h as indicated. (B) Western blot analysis of phospho‐S6K, total S6K, phospho‐S6, total S6, and actin of EV and NICD cells incubated in the absence of glutamine during 72 h as indicated. (C) Western blot analysis of NICD, phospho‐S6, total S6, and actin of CUTLL1 and H33HJ‐JA1 cells incubated either in the presence or the absence of GSI (DAPT 10 µm) for 72 h as indicated. (D) Western blot analysis of phospho‐S6K, total S6K, phospho‐S6, total S6, and actin of glutamine‐starved EV and NICD cells incubated in the presence or the absence of RAP (100 nm) for 72 h as indicated. (E‐F) Cell viability, as estimated by trypan blue assay, of EV (E) and NICD (F) cells incubated either in the presence or the absence of glutamine (Q) and RAP (100 nm) for the indicated time. (G) Late apoptotic cell percentage of EV and NICD cells incubated either in the presence or in the absence of glutamine (Q) and RAP (100 nm) for 72 h. Late apoptotic cell percentage was quantified using flow cytometry analysis of 7‐AAD and annexin V content. (H) Infiltration of leukemic human (CD3 positive) cells in the bone marrow of mice implanted with NICD H33HJ‐JA1 cells, fed either in the presence or in the absence of glutamine (Q), and treated with temsirolimus (4 mg·kg^−1^, twice a week), a derivative of RAP (20 mice/group). Graphs show mean values ± SEM (*n* ≥ 3, *P < 0.05). Two‐way ANOVA followed by Bonferroni's comparison as a *post hoc* test was used to evaluate the statistical difference between more than two groups.

Glutamine metabolism is very closely connected to mTORC1, a master controller of cell growth and metabolism [7, 18], and a therapeutic target for cancer [19]. Hence, we investigated the potential connection between Notch1 and the activation of mTORC1. We observed almost no differences in mTORC1 activation (as determined by the phosphorylation status of the mTORC1 downstream targets S6K and S6) between Notch1‐positive and Notch1‐negative cells in glutamine‐rich conditions (Fig. S3c‐d). However, mTORC1 activity was highly sustained during glutamine restriction in Notch1‐positive cells, while mTORC1 was strongly inhibited in Notch1‐negative cells (Fig. 7A,B). In agreement with this result, Notch1 inhibition using GSI strongly reduced mTORC1 activity specifically in Notch1‐positive cells (Fig. 7C), indicating that Notch1 is the main determinant of mTORC1 activity in Notch1‐positive cells. Conversely, mTORC1 inhibition using RAP induced a strong arrest of cell proliferation in Notch1‐positive cells, but it had almost no effect on Notch1‐negative cells (Fig. S3e‐f). Thus, Notch1‐positive cells are not only addicted to glutamine, but also require mTORC1 activation to sustain cell proliferation, a requirement not seen in Notch1‐negative cells. We next investigated if glutamine starvation and RAP treatment exerted a synergistic effect in the induction of cell death in Notch1‐positive leukemia. Indeed, although RAP treatment efficiently inhibited mTORC1 both in Notch1‐positive and Notch1‐negative T‐ALL cells (Figs 7D and S3g), it induced a further decrease of cell viability only in Notch1‐positive cells during glutamine‐restrictive conditions (Fig. 7E,F and S3h‐i). This decrease of cell viability was accompanied by a significant increase in apoptosis (Figs 7G and S4a‐c). Finally, to validate the physiological relevance of the treatment combining RAP and glutamine restriction *in vivo*, we induced Notch1‐positive leukemia in immunodepressed mice fed with either a complete diet or with a diet without glutamine and glutamate. These mice were then co‐treated with temsirolimus (a RAP analog), and we determined infiltration of leukemic cells in the bone marrow. As shown in Fig. 7H, while temsirolimus had no significant effect in glutamine‐rich fed mice, it almost completely abolished the infiltration of Notch1‐positive leukemic cells in the bone marrow of mice fed with a glutamine‐free diet. These results validated the physiological relevance of the Notch1‐induced addiction to both glutamine and mTORC1.

## 4 Discussion

Aberrant Notch signaling has been reported to play an important role in the tumorigenesis of different types of cancer [20, 21, 22]. However, the role of Notch1 in T‐ALL metabolism is less evident. The present study about glutamine dependence of Notch1‐driven lymphoblastic leukemia showed a connection between Notch1 signaling and glutamine metabolism. Indeed, the upregulation of Notch1 signaling in T‐ALL induced apoptotic cell death upon glutamine withdrawal with an increase in the activation of apoptosis‐related proteins (cleaved PARP, cleaved caspase 3, and cleaved caspase 8), leading to a glutamine addiction phenotype. Notch1 inhibition using GSI efficiently rescued cell viability and blocked apoptosis, and conversely, Notch1 upregulation was sufficient to induce glutamine dependence in T‐ALL cells, showing that Notch1 was both necessary and sufficient for glutamine addiction. Moreover, we confirmed this phenotype also *in vivo* using mouse models, as we observed that specifically Notch1‐positive leukemia was unable to progress in mice fed with a glutamine‐free diet. Finally, our results showed that Notch1‐induction increased GLS expression at the mRNA level. In parallel, Notch1 blocked the accumulation of GS in glutamine‐free conditions by enhancing the proteasomal degradation of GS, ultimately responsible for the addiction to glutamine.

Mechanistically, how Notch1 induces the proteasomal degradation of GS is still unclear. Recently, it was reported that glutamine induces the degradation of GS through the activity of the cullin‐RING ubiquitin ligase 4 (CRL4) complex. Thus, under glutamine‐rich conditions GS is acetylated at lysines 11 and 14 by p300, which allows its interaction with CRL4 (CRBN), and the subsequent ubiquitination and degradation of GS by the proteasome [17]. How Notch1 interferes with this system by enhancing the acetylation of GS by p300 or increasing the activity of CRL4 (CRBN) remains an open question.

Although glutamine addiction has been reported in many cancer types [23, 24, 25], to the best of our knowledge this is the first time that glutamine addiction has been reported as a consequence of Notch1 activation in T‐ALL. Previously, Herranz *et al*. [10], showed that glutaminolysis is a critical pathway for leukemia cell growth downstream of Notch1 and a key determinant of the response to anti‐Notch1 therapies *in vivo*. This work showed that, mechanistically, the inhibition of Notch1 induces glutaminolysis inhibition and triggers autophagy supporting leukemic survival and cell growth by recycling essential metabolites required for leukemic cell metabolism. Following a different approach, our results confirmed the important connection between Notch1 signaling and glutamine metabolism. However, and in contrast with their conclusions, our results indicated that GLS inhibition did not have the same impact than glutamine depletion on cell viability in Notch1‐driven leukemia, illustrating that glutamine is essential for Notch1‐positive T‐ALL cells for reasons that exceed just glutaminolysis. This conclusion was further supported by our observation that GS degradation (and not GLS) was responsible for the glutamine addiction phenotype.

The results obtained in this study highlighted the potential involvement of glutamine restriction as a therapeutic approach for Notch1‐positive T‐ALL patients. In our model, we used a glutamine/glutamate‐free diet to fed mice‐bearing Notch1‐induced leukemia. Although the liver of mice can synthesize its own glutamine, our results showed that the blood glutamine levels in mice fed with glutamine‐free diet were actually decreased significantly compared to the normal diet. Thus, the establishment of glutamine‐free diets could be considered to apply for patients bearing Notch1‐driven leukemia. However, from the practical point of view, preparation of such a diet might be not achievable, or at least not affordable. Alternatively, the use of l‐asparaginase could be envisioned in this situation. l‐asparaginase is an enzyme which catalyzes the conversion of l‐asparagine to aspartic acid and ammonia. However, it has been reported that l‐asparaginase does not only degrade l‐asparagine, but also has a GLS activity although with lower affinity and lower maximal rate, leading to decreased levels of glutamine in blood [26, 27, 28]. Considering that this type of treatment is already available for drug administration, it could be contemplated for the treatment of T‐ALL patients carrying Notch1 mutations.

Finally, our results showed that a treatment combining glutamine depletion with the mTORC1 inhibitor reduces the viability specifically of Noch1‐driven leukemic cells. The mechanistic link observed between Notch1 and mTORC1 through glutaminolysis seems to operate as a mechanism to potentiate cell growth and leukemia proliferation, sustaining high glutamine catabolism and high mTORC1 activity. Targeting this axis by attacking to main points (glutamine availability and mTORC1 activation) could be envisioned as a potential therapy to treat Notch1‐positive T‐ALL patients.

## Conflict of interest

The authors declare no conflict of interest.

## Author contributions

RVD conceived the project. TLN, MJN, ST, and RVD designed experiments. TLN, MJN, ST, MT, CB, OG, JMP, BR, SvL, JMF, ER, HRR, MP, MB, IR, JC, BU, and PF performed experiments. TLN, MJN, ST, MT, CB, OG, and RVD analyzed data. RVD, PS, PSM, FP, MLT, and AMK secured funding. RVD, TLN, and MJN wrote the manuscript.

## Supporting information


**Fig. S1.** Glutamine sustains TCA cycle in T‐ALL cells.
**Fig. S2.** Notch1 activation/upregulation correlated with glutamine addiction in T‐ALL cells.
**Fig. S3.** mTORC1 inhibition synergizes with glutamine starvation to reduce cell proliferation in Notch1‐positive T‐ALL.
**Fig. S4.** mTORC1 inhibition synergizes with glutamine starvation to induce cell death in Notch1‐positive T‐ALL.

## Data Availability

The authors declare that all the data supporting the findings of this study are available within the article and its Supporting information files and from the corresponding author upon reasonable request.
