# Supplementary figures and images for: Downregulation of Glutamine Synthetase, not glutaminolysis, is responsible for glutamine addiction in Notch1‐driven acute lymphoblastic leukemia
Source: Mol Oncol. 2021 Feb 13;15(5):1412–31. doi: 10.1002/1878-0261.12877 (PMC8096784; doi:10.1002/1878-0261.12877)

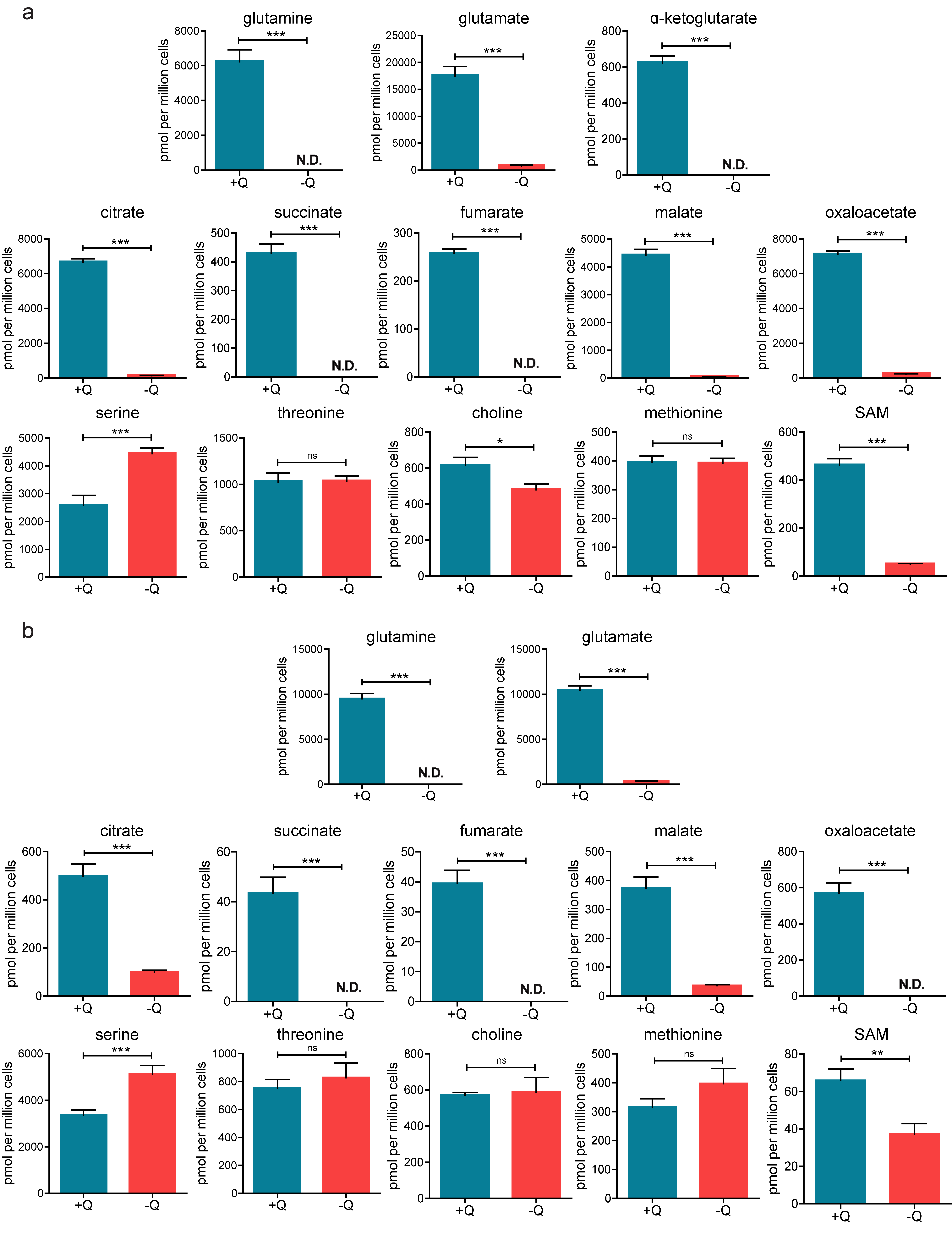

Supplement: Supplementary file 1 — Fig. S1. Glutamine sustains TCA cycle in T‐ALL cells. Fig. S2. Notch1 activation/upregulation correlated with glutamine addiction in T‐ALL cells. Fig. S3. mTORC1 inhibition synergizes with glutamine starvation to reduce cell proliferation in Notch1‐positive T‐ALL. Fig. S4. mTORC1 inhibition synergizes with glutamine starvation to induce cell death in Notch1‐positive T‐ALL. [file MOL2-15-1412-s001.zip › mol212877-sup-0001-FigS1.tif]

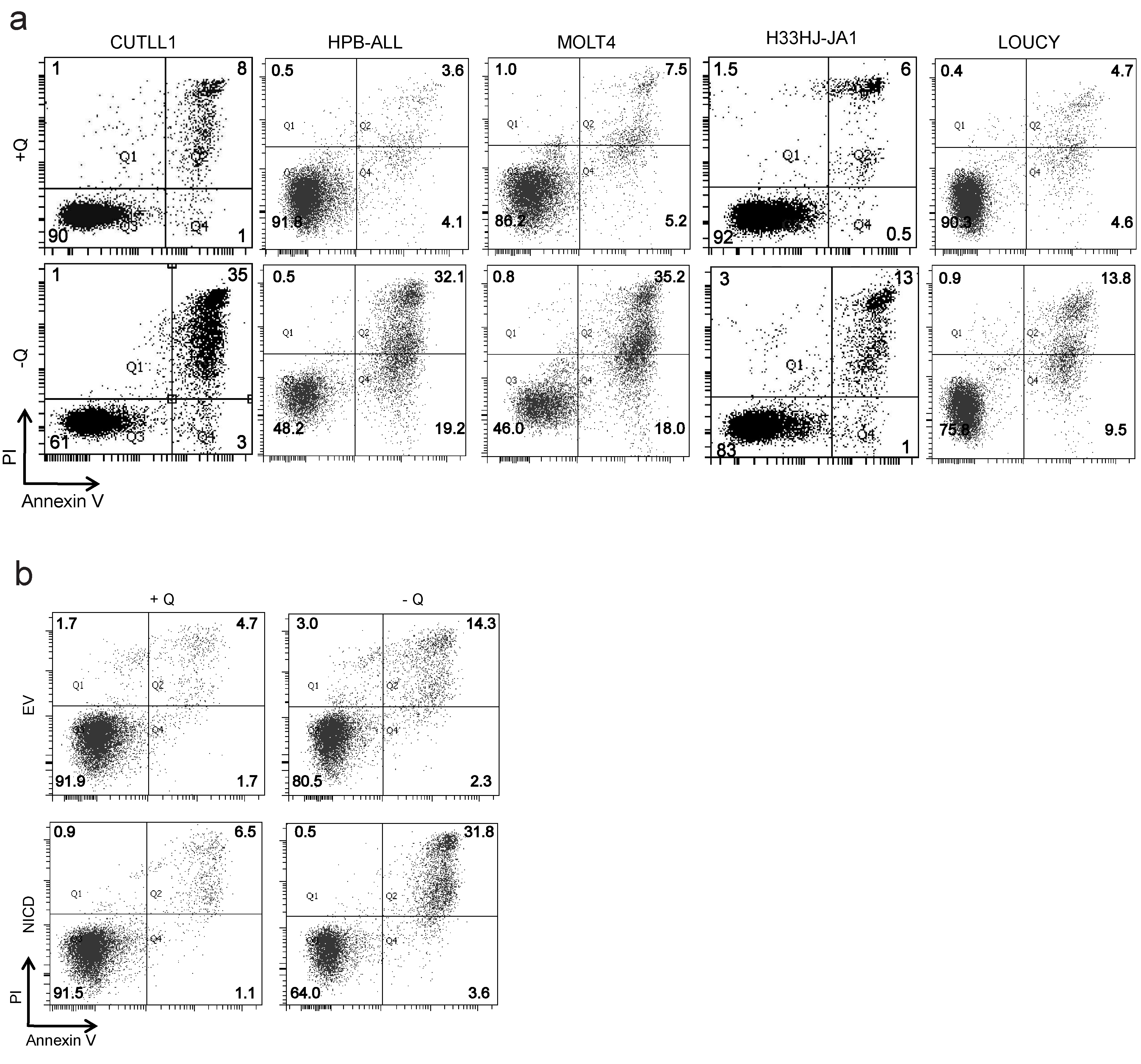

Supplement: Supplementary file 1 — Fig. S1. Glutamine sustains TCA cycle in T‐ALL cells. Fig. S2. Notch1 activation/upregulation correlated with glutamine addiction in T‐ALL cells. Fig. S3. mTORC1 inhibition synergizes with glutamine starvation to reduce cell proliferation in Notch1‐positive T‐ALL. Fig. S4. mTORC1 inhibition synergizes with glutamine starvation to induce cell death in Notch1‐positive T‐ALL. [file MOL2-15-1412-s001.zip › mol212877-sup-0002-FigS2.tif]

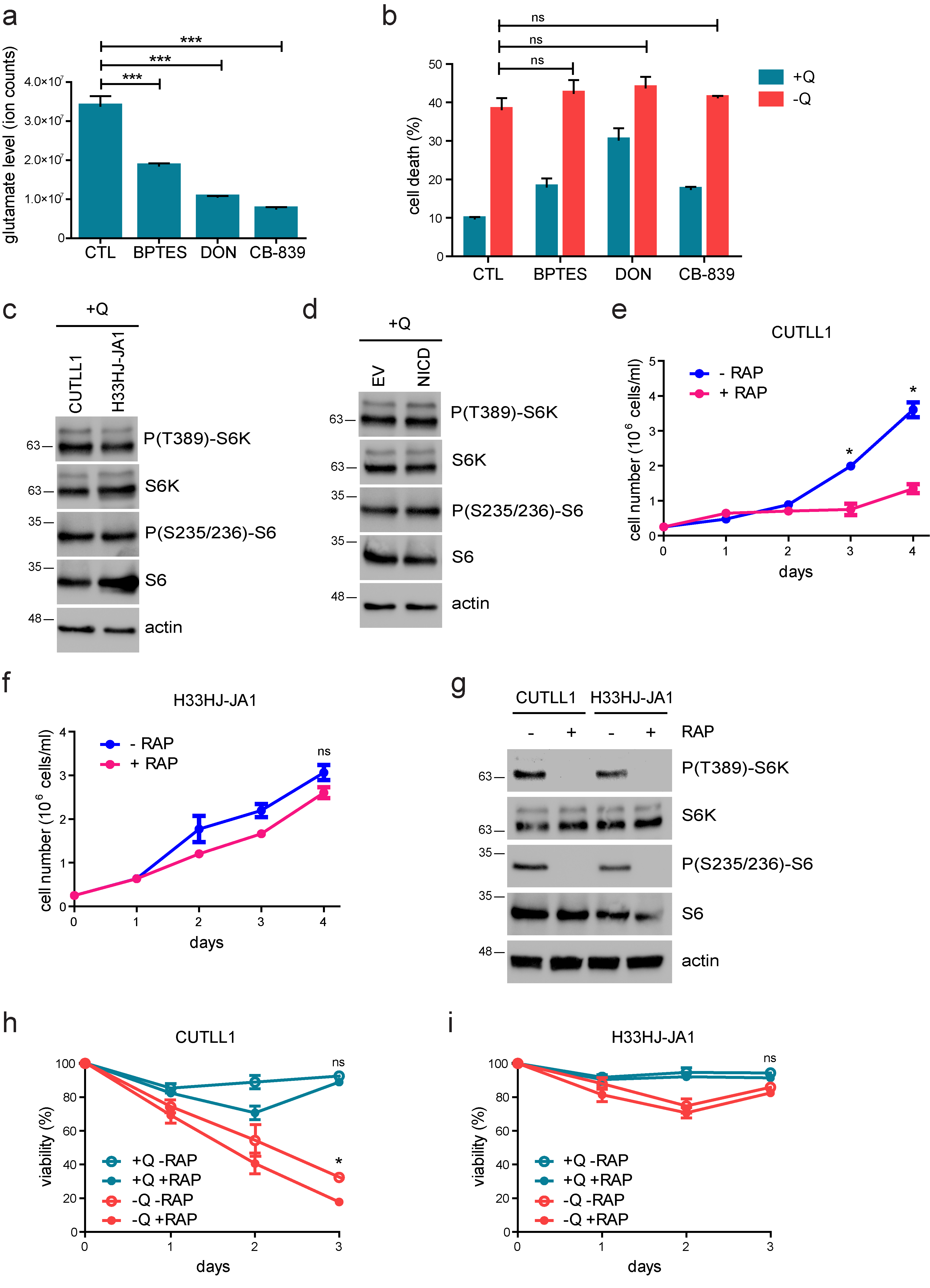

Supplement: Supplementary file 1 — Fig. S1. Glutamine sustains TCA cycle in T‐ALL cells. Fig. S2. Notch1 activation/upregulation correlated with glutamine addiction in T‐ALL cells. Fig. S3. mTORC1 inhibition synergizes with glutamine starvation to reduce cell proliferation in Notch1‐positive T‐ALL. Fig. S4. mTORC1 inhibition synergizes with glutamine starvation to induce cell death in Notch1‐positive T‐ALL. [file MOL2-15-1412-s001.zip › mol212877-sup-0003-FigS3.tif]

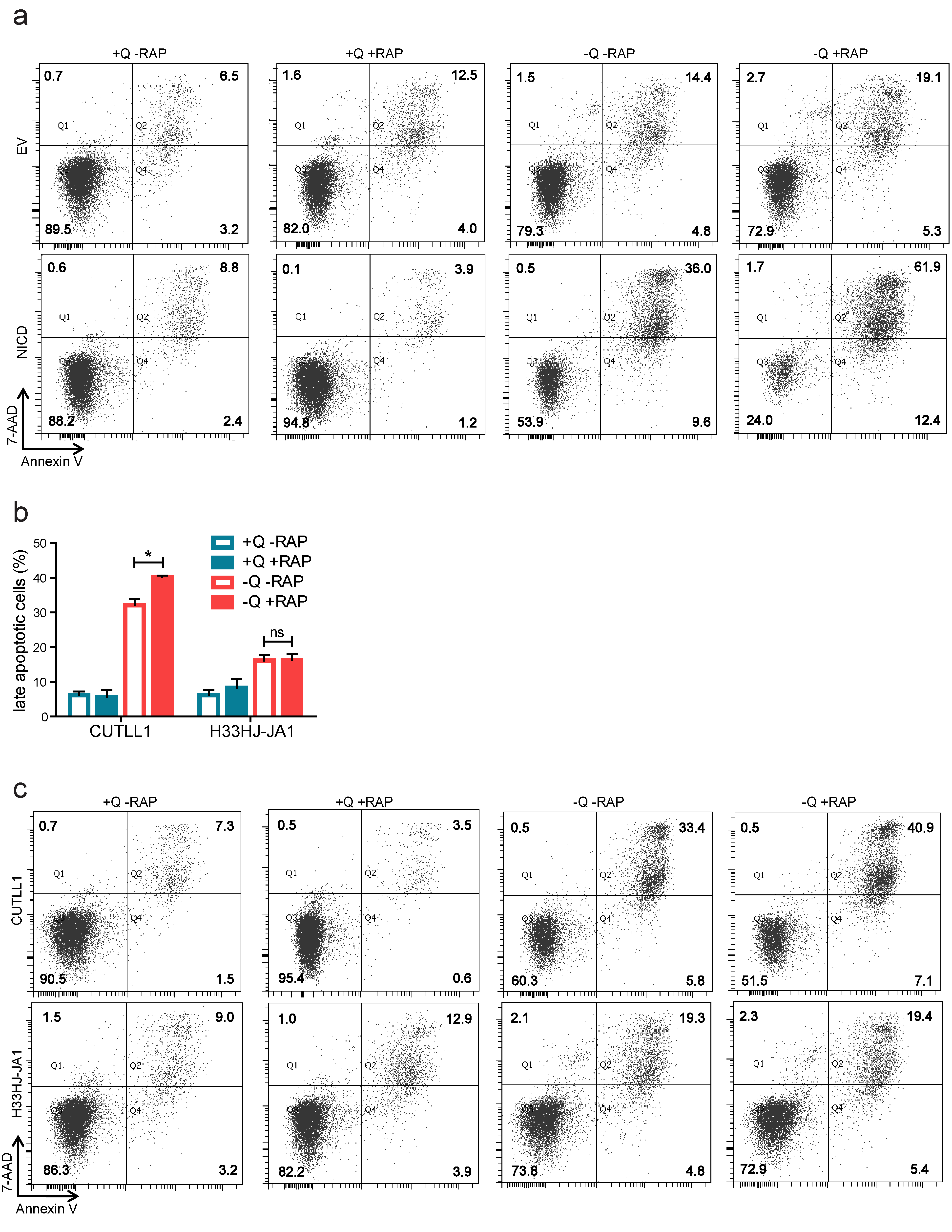

Supplement: Supplementary file 1 — Fig. S1. Glutamine sustains TCA cycle in T‐ALL cells. Fig. S2. Notch1 activation/upregulation correlated with glutamine addiction in T‐ALL cells. Fig. S3. mTORC1 inhibition synergizes with glutamine starvation to reduce cell proliferation in Notch1‐positive T‐ALL. Fig. S4. mTORC1 inhibition synergizes with glutamine starvation to induce cell death in Notch1‐positive T‐ALL. [file MOL2-15-1412-s001.zip › mol212877-sup-0004-FigS4.tif]
