# Supplementary material for: Downregulation of Glutamine Synthetase, not glutaminolysis, is responsible for glutamine addiction in Notch1‐driven acute lymphoblastic leukemia
Source: Mol Oncol. 2021 Feb 13;15(5):1412–31. doi: 10.1002/1878-0261.12877 (PMC8096784; doi:10.1002/1878-0261.12877)
Supplement: Supplementary file 1 — Fig. S1. Glutamine sustains TCA cycle in T‐ALL cells. Fig. S2. Notch1 activation/upregulation correlated with glutamine addiction in T‐ALL cells. Fig. S3. mTORC1 inhibition synergizes with glutamine starvation to reduce cell proliferation in Notch1‐positive T‐ALL. Fig. S4. mTORC1 inhibition synergizes with glutamine starvation to induce cell death in Notch1‐positive T‐ALL. [file MOL2-15-1412-s001.zip › mol212877-sup-0005-Legends.doc]

**Supplementary Data**

**Downregulation of Glutamine Synthetase, not glutaminolysis, is responsible for glutamine addiction in Notch1-driven acute lymphoblastic leukemia**

Tra Ly Nguyen, Marie-Julie Nokin, Silvia Terés, Mercedes Tomé, Clément Bodineau, Oriane Galmar, Jean-Max Pasquet, Benoit Rousseau, Sebastian van Liempd, Juan Manuel Falcon-Perez, Elodie Richard, Elodie Muzotte, Hamid-Reza Rezvani, Muriel Priault, Marion Bouchecareilh, Isabelle Redonnet-Vernhet, Julien Calvo, Benjamin Uzan, Françoise Pflumio, Patricia Fuentes, Maria L. Toribio, Abdel-Majid Khatib, Pierre Soubeyran, Piedad del Socorro Murdoch and Raúl V. Durán

**Supplementary Figure legends**

**Supplementary Figure S1. Glutamine sustains TCA cycle in T-ALL cells**. **a**-**b**, Targeted metabolomic analysis of H33HJ-JA1 (a) and CUTLL1 (b) cells incubated either in the presence (+Q) or absence (-Q) of glutamine for 24h. Metabolite concentration was analysed by mass spectrometry. Graphs show mean values ± S.E.M. (n=3). “ND” indicates values below the detection level. *t*-test analysis was used to evaluate the statistical difference between two groups.

**Supplementary Figure S2. Notch1 activation/upregulation correlated with glutamine addiction in T-ALL cells**. **a**, CUTLL1, HBP-ALL, MOLT4, H33HJ-JA1, and LOUCY cells were incubated either in the presence (+Q) or absence (-Q) of glutamine for 72h. Late apoptotic cell percentage was estimated through flow cytometry analysis of propidium iodide (PI) and annexin V content. **b**, EV and NICD H33HJ-JA1 cells were incubated either in the presence (+Q) or absence (-Q) of glutamine for 72h. Late apoptotic cell percentage was estimated by flow cytometry analysis of propidium iodide (PI) and annexin V content. Graphs show mean values ± S.E.M. (n≥3, * *p*<0.05).

**Supplementary Figure S3. mTORC1 inhibition synergizes with glutamine starvation to reduce cell proliferation in Notch1-positive T-ALL**. **a**, Glutamate level in CUTLL1 incubated in presence of BPTES (30 µM), DON (30 µM) or CB-839 (10 µM) for 72 hours. **b**, Cell death estimation of CUTLL1 cells incubated either in the presence or absence of glutamine (Q) and BPTES (30µM), DON (30 µM) or CB-389 (10 µM) during 72h as indicated. **c**, CUTLL1 and H33HJ-JA1 cells were incubated in a complete medium for 24h. Cell extracts were collected and levels of phospho-S6K, total S6K, phospho-S6, total S6, and actin were estimated by western blot. **d**, EV and NICD H33HJ-JA1 cells were incubated in a complete medium for 24h. Cell extracts were collected and levels of phospho-S6K, total S6K, phospho-S6, total S6, and actin were estimated by western blot. **e-f**, H33HJ-JA1 (e) and CUTLL1 (f) cells were incubated in a complete medium either in the presence or absence of rapamycin (RAP, 100nM). Cell concentration was determined using a cell counter. **g**, Glutamine-starved CUTLL1 and H33HJ-JA1 cells where incubated either in the presence or the absence rapamycin (RAP, 100nM) during 72h as indicated. Cell extracts were collected and levels of phospho-S6K, total S6K, phospho-S6, total S6, and actin were estimated by western blot.  **h-i**, CUTLL1 (h) and H33HJ-JA1 (i) cells were incubated either in the presence (+Q) or absence (-Q) of glutamine and rapamycin (RAP, 100nM) during the indicated time. Cell viability was estimated using a trypan blue assay. Graphs show mean values ± S.E.M. (n≥3, * *p*<0.05). One-way or two-way ANOVA followed by Bonferroni’s comparison as a post hoc test was used to evaluate the statistical difference between more than two groups. *t*-test analysis was used to evaluate the statistical difference between two groups.

**Supplementary Figure S4. mTORC1 inhibition synergizes with glutamine starvation to induce cell death in Notch1-positive T-ALL**. **a**, Apoptosis induction was estimated using flow cytometry analysis of 7-AAD and annexin V content in EV and NICD cells incubated either in the presence or absence of glutamine (Q) and rapamycin (RAP, 100nM) for 72h (related to Figure 7g). **b-c**, CUTLL1 and H33HJ-JA1 cells were incubated either in the presence or absence of glutamine (Q) and rapamycin (RAP, 100nM) during 72h as indicated. Then late apoptotic cell percentage was estimated through flow cytometry analysis of 7-AAD and annexin V content. Graphs show mean values ± S.E.M. (n≥3, * *p*<0.05). Two-way ANOVA followed by Bonferroni’s comparison as a post hoc test was used to evaluate the statistical difference between more than two groups.
